# Engineering Active Interfaces on the Surface of Porous Single-Crystalline TiO_2_ Monoliths for Enhanced Catalytic Activity and Stability

**DOI:** 10.34133/research.0579

**Published:** 2025-01-14

**Authors:** Huang Lin, Cong Luo, Fangyuan Cheng, Kui Xie

**Affiliations:** ^1^ Key Laboratory of Design & Assembly of Functional Nanostructures, Fujian Institute of Research on the Structure of Matter, Chinese Academy of Sciences, Fuzhou, Fujian 350002, China.; ^2^ Fujian Science & Technology Innovation Laboratory for Optoelectronic Information of China, Fuzhou, Fujian 350108, China.; ^3^ University of Chinese Academy of Sciences, Beijing 100049, China.; ^4^ Fujian College, University of Chinese Academy of Sciences, Fuzhou, Fujian 350108, China.; ^5^ School of Mechanical Engineering, Shanghai Jiao Tong University, Shanghai 200240, China.

## Abstract

The engineering design and construction of active interfaces represents a promising approach amidst numerous initiatives aimed at augmenting catalytic activity. Herein, we present a novel approach to incorporate interconnected pores within bulk single crystals for the synthesis of macroscopic porous single-crystalline rutile titanium oxide (R-TiO_2_). The porous single crystal (PSC) R-TiO_2_ couples a nanocrystalline framework as the solid phase with pores as the fluid phase within its structure, providing unique advantages in localized structure construction and in the field of catalysis. We successfully construct well-defined Ni cluster/TiO_2_ active interfaces by directly confining Ni clusters on the continuous lattice surface of PSC R-TiO_2_. We confirm that the lattice oxygen connected to the Ni clusters exhibits exceptional activation capability at temperatures close to room temperature compared to the pure phase PSC R-TiO_2_ monoliths. The PSC Ni/TiO_2_ catalyst demonstrates complete CO oxidation and stable catalytic performance during continuous operation in air at ~80 °C for 200 h.

## Introduction

Porous single crystals (PSCs) at the macroscale are considered a novel type of inorganic porous material that combines the characteristics of single crystals and porosity [1]. PSCs feature internal pores capable of carrying fluid phases in a 3-dimensional interconnected porous structure, while the continuous framework acts as the solid phase, exhibiting a long-range ordered single-crystal state in the lattice [2]. The traditional concept of bulk single-crystal materials generally refers to solid-phase materials with dimensions reaching centimeters or larger, characterized by the ordered arrangement and oriented repetitive combination of components such as atoms, ions, and molecules in 3-dimensional space [3,4]. Due to the high symmetry and ordered structure of single crystals, it is commonly recognized that the performance of materials can be substantially enhanced when they are in a single-crystal state [5]. Porous materials are typically materials with pores that are directionally designed to achieve specific functions [6,7]. Catalytic materials with porous structures can not only reduce effective mass density but also increase specific surface area, thereby expanding the capacity of catalytic reactions [8,9]. Customizing pores in bulk single crystals is a promising approach to creating novel porous materials. This material is characterized by the organic integration of nano-single-crystal frameworks and pore structures while maintaining crystal structure integrity independent of grain boundaries Therefore, this offers invaluable opportunities to enhance the overall performance of materials by altering their physical characteristics by leveraging the unique properties of crystals and manipulating customized growth conditions [10].

The high stability and catalytic activity of TiO_2_ make it widely recognized as a crucial catalyst, leading to substantial research interest in its applications such as gas-phase catalysis, energy conversion, and organic pollutant removal [11,12]. Rutile-phase TiO_2_ (R-TiO_2_), the most stable structure in titanium dioxide, exhibits strong catalytic activity, high stability, good resistance to poisoning, and low cost, making it one of the most promising heterogeneous catalysts with great development potential [13]. However, the majority of reported R-TiO_2_ exists in nano-powder form and their short-range ordered lattice structure limits their potential applications in catalysis [14]. To address this limitation and enhance its catalytic efficacy substantially, PSC R-TiO_2_ monoliths have been developed. These monoliths combine porous materials with single crystals to create homogeneous 3-dimensional pathways and notably improve catalysis activity [15,16].

The utilization of oxide scaffolds with oxygen defects in the metal–oxide interface system holds great promise as a modification method in metal catalysts, enhancing lattice oxygen activation during the reaction [17–19]. For instance, the utilization of Pt supported on Al_2_O_3_ enables to complete oxidation of CO at low temperatures due to the synergistic effect between Pt/Al_2_O_3_ sites [20,21]. The metal–oxide interface system between Au and TiO_2_ support was investigated by Yuan et al. [22] using in situ spherical aberration-corrected transmission electron microscopy (Cs-STEM). At the atomic scale, the presence of the Au/TiO_2_ interface system can effectively enhance the catalytic performance of TiO_2_ during CO oxidation reaction. However, the development of loaded precious metal catalysts has been somewhat constrained by the drawbacks of their high cost and limited reserves. The utilization of transition metals holds substantial potential in the modification of catalysts [23–25]. The field of catalysis has witnessed substantial attention toward transition metal Ni, with scholars exploring the loading techniques to enhance its catalytic activity in reactions such as volatile organic compound oxidation [26] and alkylphosphine compound hydrogenation [27]. The interaction between transition metal Ni and catalyst promotes its advantages in terms of catalytic oxidation activity and stability. By employing nickel as the catalyst instead of precious metals to create a metal–oxide interface system between Ni clusters and TiO_2_, it is possible to prepare a highly active and stable catalyst for CO oxidation reaction.

In this work, we fabricated the metal–oxide interface system by growing centimeter-scale PSC R-TiO_2_ monoliths and depositing atomic-layer Ni clusters on its long-range ordered lattice surface using atomic layer deposition (ALD) techniques. The charge transfer between Ni and the surface lattice oxygen sites on R-TiO_2_ at the Ni cluster/TiO_2_ interface system promotes the chemical adsorption and activation of CO molecules while simultaneously activating adjacent surface lattice oxygen in TiO_2_ due to distortion caused by Ni clusters/TiO_2_ at the interface. This dual activation provides an advantage for efficient and durable CO oxidation reaction. We evaluated the performance of PSC TiO_2_ loading with Ni cluster (PSC Ni/TiO_2_) catalyst, which exhibited complete oxidation of CO at low temperature (80 °C) with substantial catalytic stability during 200 h of continuous reaction. The creation of PSC R-TiO_2_ and design of this interface system provide a new pathway for synthesizing and applying large-pore single-crystal materials.

## Results

**Fig. 1.**
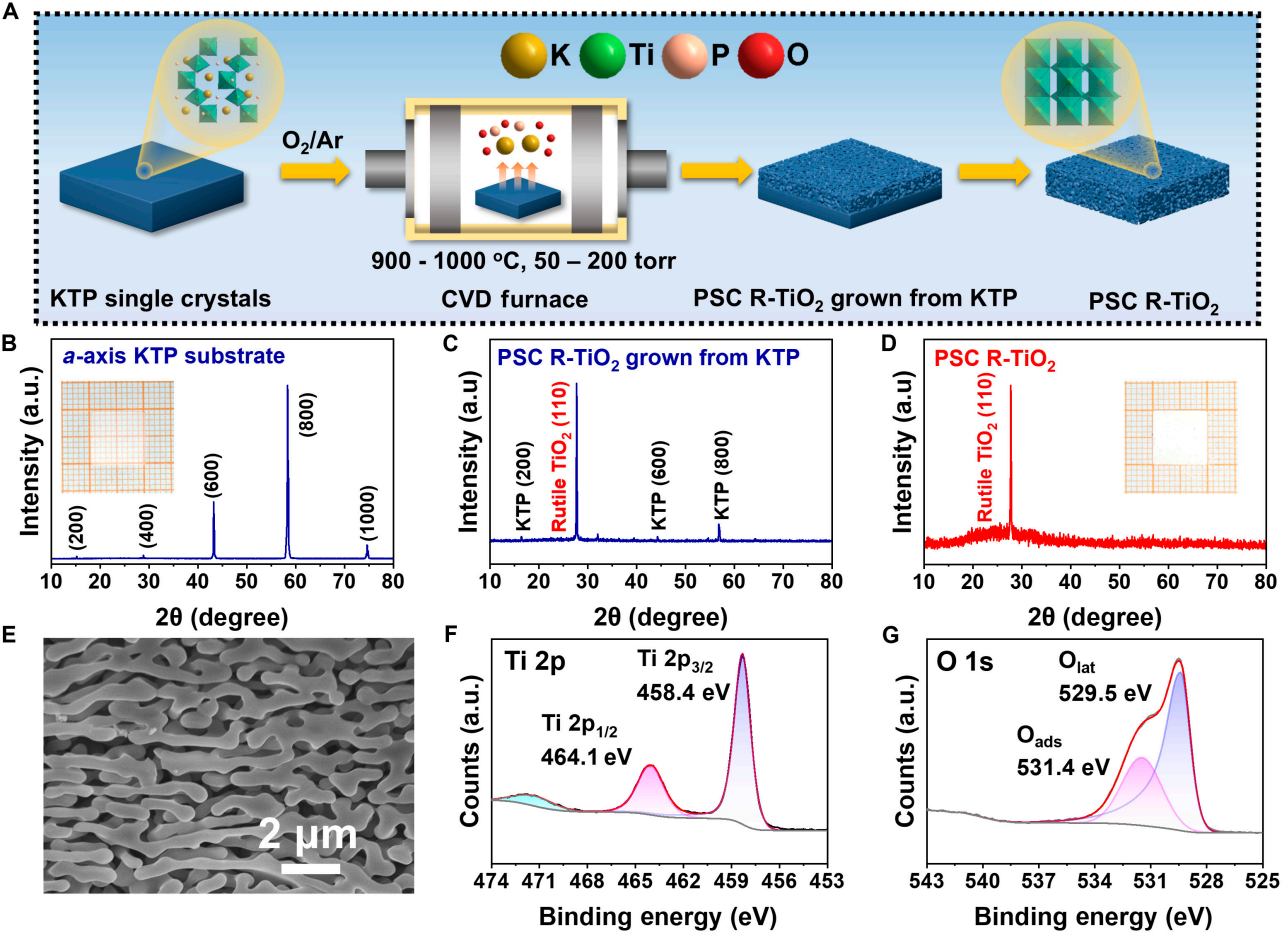
Growth of PSC R-TiO_2_ monoliths from KTP. (A) Preparation of PSC R-TiO_2_. (B to D) XRD patterns of PSC R-TiO_2_ at various growth stages. The inset images are digital photographs of KTP (B) and PSC R-TiO_2_ (D) monolithic samples measuring 10 mm × 10 mm × 0.5 mm. (E) SEM image of PSC R-TiO_2_ . (F and G) XPS spectra of PSC R-TiO_2_.

The growth of KTiOPO_4_ (KTP) single crystals is achieved using a molten-salt growth method that utilizes molybdates as the flux [28]. The synthesis and growth process of PSC R-TiO_2_ single crystal and experimental setup are illustrated in Fig. 1A and Fig. S1. In brief, we cultivate KTP single crystals and divide them into substrates measuring 10 mm on each side and having a thickness of 0.5 mm. After the polishing process, we subjected the KTP with (100) facet to an O_2_/Ar atmosphere ranging from 50 to 400 torr, with a controlled flow rate of 50 to 200 sccm (standard cubic centimeter per minute), while maintaining a temperature range of 900 to 1,000 °C. This approach aimed to cultivate porous TiO_2_ single crystals through a high-temperature lattice reconstruction technique, as illustrated in Fig. S2. The K and P atoms migrate away from the lattice structure of KTP, causing the left Ti–O structures to undergo a reconstructive process and form an ordered lattice. As a result, PSC R-TiO_2_ monoliths are formed, possessing dimensions comparable to the substrates. The KTP eventually undergoes a transformation into centimeter-scale single crystals of porous R-TiO_2_. The lattice channels in KTP facilitate the removal of P/K, thereby promoting the growth of PSC R-TiO_2_ monoliths.

The reconstruction of the Ti–O framework results in approximately 70.0% porosity within the porous architectures. Due to the different lattice constants of substrate and epitaxial layer, lattice mismatch will occur during the growth of TiO_2_ on the KTP substrate. The lattice constants of the substrate are denoted as *a*_*s*_, while those of the epitaxial layer are represented by *a*_*f*_. The resulting lattice mismatch δ can be calculated using Eq. 1 [29]:

$$\delta =\frac{\left|a_{s}-a_{f}\right|}{a_{f}}\;$$ (1)

A reduction in the lattice mismatch rate leads to a decrease in the lattice strain grown on the substrate, thereby enhancing the degree of orientation correlation [30]. After calculation, the lattice mismatch ratio of (100) facet KTP single crystal corresponding to R-TiO_2_ is found to be as low as 1.24%. Therefore, the (100) facet single-crystal KTP with the lowest lattice mismatch rate is an ideal substrate for the preparation of R-TiO_2_. The lattice structure of R-TiO_2_ prepared on various crystal facets was characterized using x-ray diffraction analysis (XRD) and scanning electron microscopy (SEM), as illustrated in Figs. S3 to S5. The TiO_2_ synthesized using *a*-axis oriented KTP exhibits enhanced crystallinity and uniform porosity, as evidenced by the observations. Due to its minimal lattice mismatch rate, the *a*-axis oriented KTP selectively undergoes transformation into R-TiO_2_ at elevated temperatures while minimizing the occurrence of lattice defects [31]. The prepared materials were characterized by XRD to determine their material phase and the orientation of crystal facet. The XRD pattern of the synthesized KTP closely matches the diffraction peak of the corresponding crystal surface of the standard material, as illustrated in Fig. 1B to D, indicating exceptional crystallinity of the precursor KTP crystal prepared. The diffraction peak of porous TiO_2_ single crystal is precisely matched with the characteristic peak of known rutile type (110) facet TiO_2_ crystal (PDF#88-1174). The successful preparation of centimeter-scale rutile phase single-crystal TiO_2_ with high crystallinity has been demonstrated through the utilization of KTP single crystal as the mother crystal and employing a high-temperature lattice reconstruction technique.

The SEM is utilized to analyze the microstructure and morphology of the prepared samples. The prepared PSC R-TiO_2_ exhibits uniform 3-dimensional pores while maintaining its single-crystal property, thereby substantially enhancing the specific surface area and providing more adsorption sites for improved performance. The porous structure displays a uniform distribution of macropores, as shown in Fig. 1E, and Fig. S7 illustrates the PSC TiO_2_ grown from different crystal facets of KTP, revealing consistent presence of uniform porous structures. However, considering that the R-TiO_2_ obtained from the (100) facet of KTP theoretically exhibits minimal defects, we opted to employ R-TiO_2_ prepared from the (100) facet of KTP for subsequent tests. We utilize x-ray photoelectron spectroscopy (XPS) for the examination of surface chemistry states linked to the PSC R-TiO_2_ structure illustrated in Fig. 1F and G. The Ti 2p spectrum of PSC R-TiO_2_ exhibits 2 distinct peaks at 458.4 and 464.1 eV, corresponding to the Ti 2p_3/2_ (TiO_2_) and Ti 2p_1/2_ (TiO_2_) states, respectively. This observation supports the prevalence of titanium element in an oxidation state of +4. The detailed O 1s spectrum exhibits prominent peaks at 530.3 and 532.4 eV, corresponding to surface lattice oxygen (O_lat_) and surface adsorbed oxygen (O_ads_), respectively [32].

**Fig. 2.**
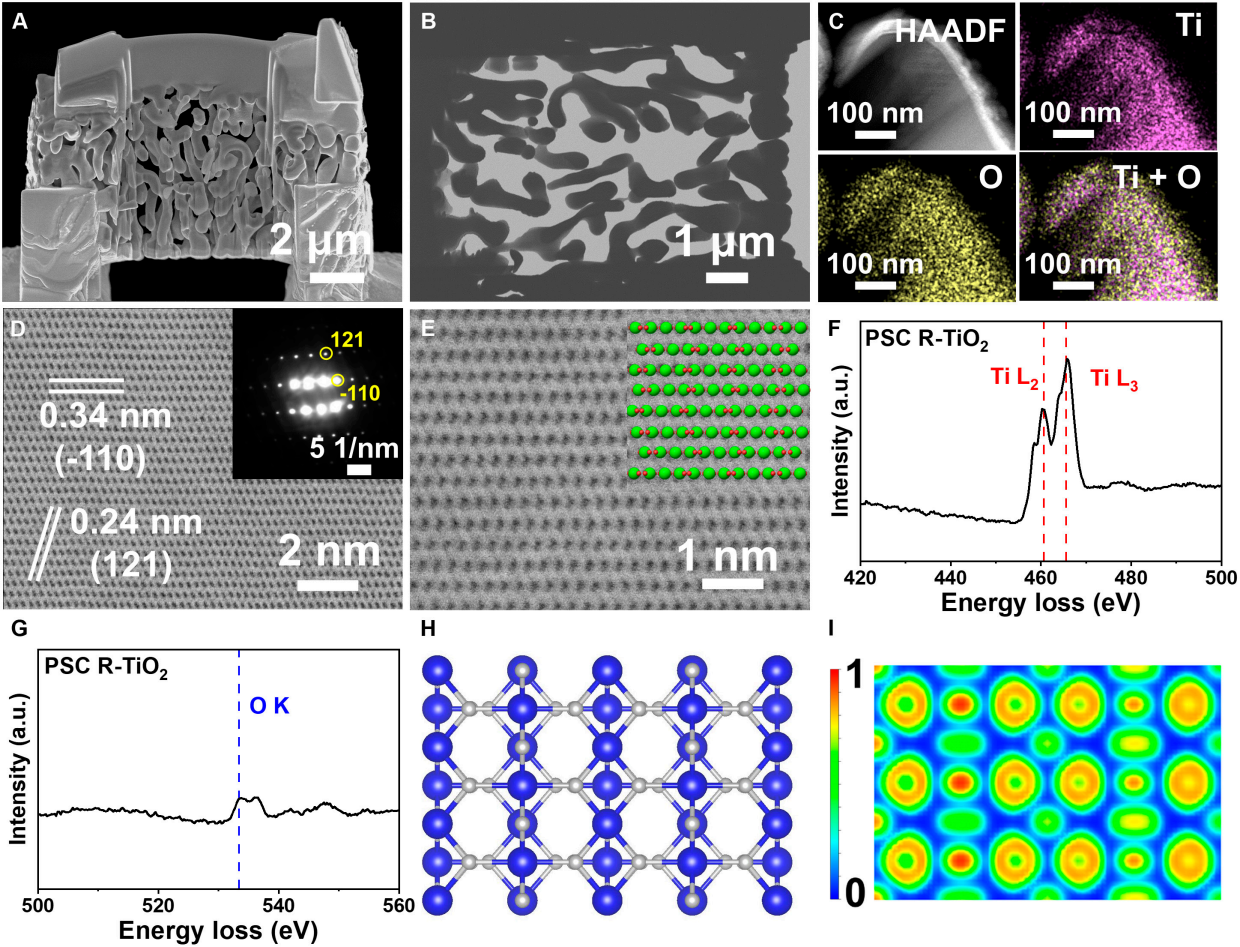
Microstructure of PSC R-TiO_2_ monoliths. (A) FIB-SEM image of PSC R-TiO_2_. (B) STEM image of the PSC R-TiO_2_ porous skeleton. (C) High-angle annular dark-field (HADDF) images of PSC R-TiO_2_. (D and E) Cs-HRTEM images and SAED pattern of PSC R-TiO_2_. The green and red spheres represent Ti and O, respectively. (F and G) EELS spectra of PSC R-TiO_2_. (H) Model of PSC (110) R-TiO_2_. (I) Charge density graph of PSC (110) R-TiO_2_.

The presence of abundant lattice oxygen in the PSC R-TiO_2_ prepared by us suggests its potential for constructing the metal–oxide interface system. The aforementioned condition provides a favorable environment for subsequent loading of Ni clusters. In Fig. S8, we employed focused ion beam SEM (FIB-SEM) and scanning TEM (STEM) to investigate the internal structure images of PSC R-TiO_2_. The structure of PSC R-TiO_2_, as depicted in Fig. 2A and B, exhibits not only surface pores with uniformity but also internal monomer pores that are uniformly distributed. The presence of interconnected pores within the porous framework substantially enhances its specific surface area, thereby enabling it to function as a porous material that offers abundant active sites and creates favorable conditions for subsequent construction of the metal–oxide interface system onto its surface. The elemental mapping of PSC R-TiO_2_, as shown in Fig. 2C and Fig. S9, provides evidence for the composition of its porous framework being TiO_2_. At the same time, it is observable in Fig. 2D and E that the prepared PSC R-TiO_2_ has excellent single-crystal property. The selected area electron diffraction (SAED) pattern of the internal framework of PSC TiO_2_ exhibits a characteristic rutile structure, as evidenced by the inset image showing a single crystal of TiO_2_, which is consistent with the XRD analysis. The periodic arrangement of atoms along the (110) and (121) directions is clearly observed in Fig. 2D. The calculated d-spacing of 0.34 nm corresponds well to the (110) facet. The atomic arrangement of rutile TiO_2_ on the high-resolution TEM (HRTEM) diagram in Fig. 2E is simulated, and its distribution exhibits a high level of agreement with that of standard rutile TiO_2_ crystals (PDF#88-1174). The electron energy loss spectra (EELS) of Ti and O are presented in Fig. 2F and G, where the peaks observed at 460, 465, and 533 eV correspond to the Ti L_3_ edge, Ti L_2_ edge, and O K edge of the standard TiO_2_ film, respectively [33,34]. The theoretical model of PSC R-TiO_2_, represented by Fig. 2H, exhibits the charge distribution of its cross section as calculated through simulation in Fig. 2I.

**Fig. 3.**
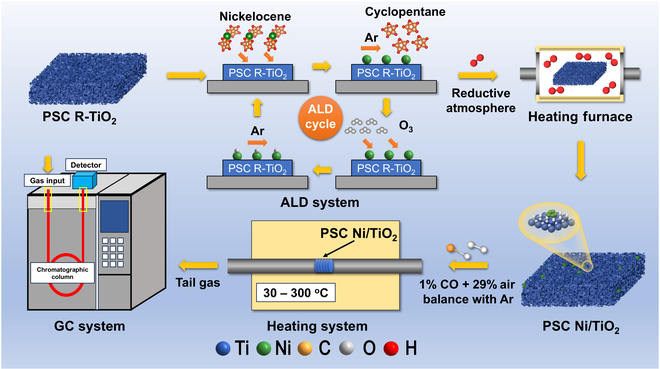
Preparation of PSC Ni/TiO_2_ and catalytic performance for CO oxidation with air of PSC Ni/TiO_2_.

**Fig. 4.**
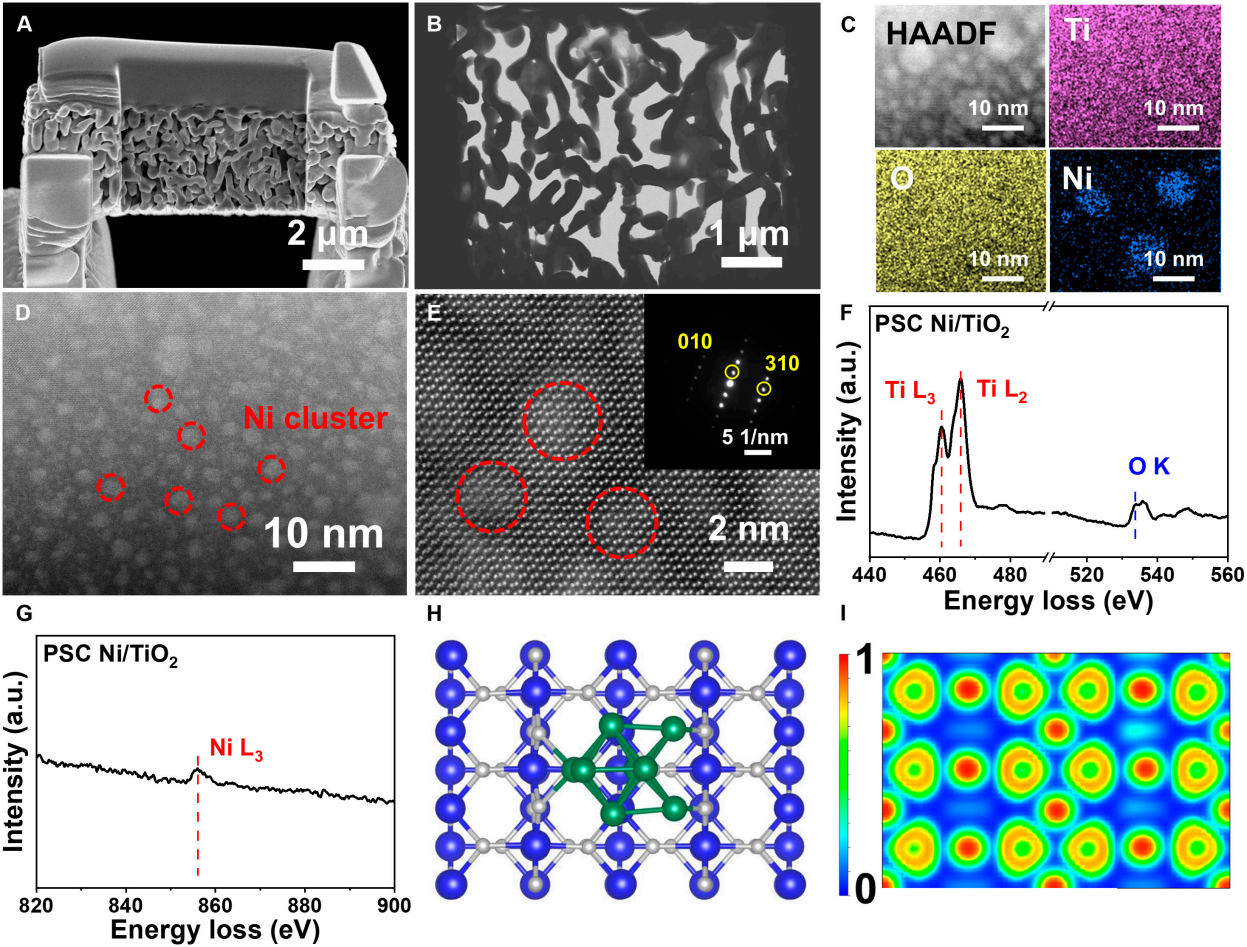
Microstructure of PSC Ni/TiO_2_ monolith. (A) FIB-SEM image of PSC Ni/TiO_2_. (B) STEM image of the PSC Ni/TiO_2_ porous skeleton. (C) HADDF image of PSC Ni/TiO_2_. (D and E) Cs-HRTEM images and SAED pattern of PSC Ni/TiO_2_. (F and G) EELS spectra of PSC Ni/TiO_2_. (H) Model of PSC Ni/TiO_2_. (I) Charge density graph of PSC Ni/TiO_2_.

We employed the ALD technique to deposit Ni clusters onto PSC R-TiO_2_ monolith, as illustrated in Fig. 3, thereby establishing a metal oxide interface system. Following multiple cycles through the ALD system, we transferred the sample into a porcelain boat and placed it in a heating furnace for reduction under a reductive atmosphere for 3 h to ensure complete conversion of all Ni species into Ni in metallic state. Through precise control of the exposure time, we successfully achieved homogeneous dispersion of nanoscale Ni clusters. The presence of these Ni clusters facilitates CO adsorption, while the enhanced activation of lattice oxygen can be attributed to the dynamic structure at the interface. The SEM and STEM images (Fig. 4A and B) demonstrate that the PSC Ni/TiO_2_ maintains the porous characteristics of TiO_2_, with the sample still exhibiting interconnected 3-dimensional pores after undergoing the ALD method. The Cs-corrected STEM (Cs-STEM) images presented in Fig. 4C to E reveal the presence of uniformly dispersed Ni clusters on the surface of PSC R-TiO_2_ monoliths. Furthermore, these images highlight the spatial arrangement of Ni clusters, indicating that the decoration with Ni effectively enhances the metal–oxide interface system. Consequently, specific regions with enhanced catalytic efficiency and activation of lattice oxygen at the interfaces are observed. Through statistical analysis of Cs-STEM images (Figs. S12 and S13) at lower magnification, it can be concluded that the majority of Ni clusters fall within the size range of 2 to 3 nm, with an average particle size of ~2.66 nm. Within this particle size range, the risk of Ni cluster crystallization is minimal. The proportion of Ni clusters larger than 5 nm is extremely low, accounting for only approximately ~3.55% of the total content. Therefore, by precisely controlling the exposure time in the ALD system, we are able to confine the size of Ni clusters within a controllable range while minimizing their degree of crystallization.

**Fig. 5.**
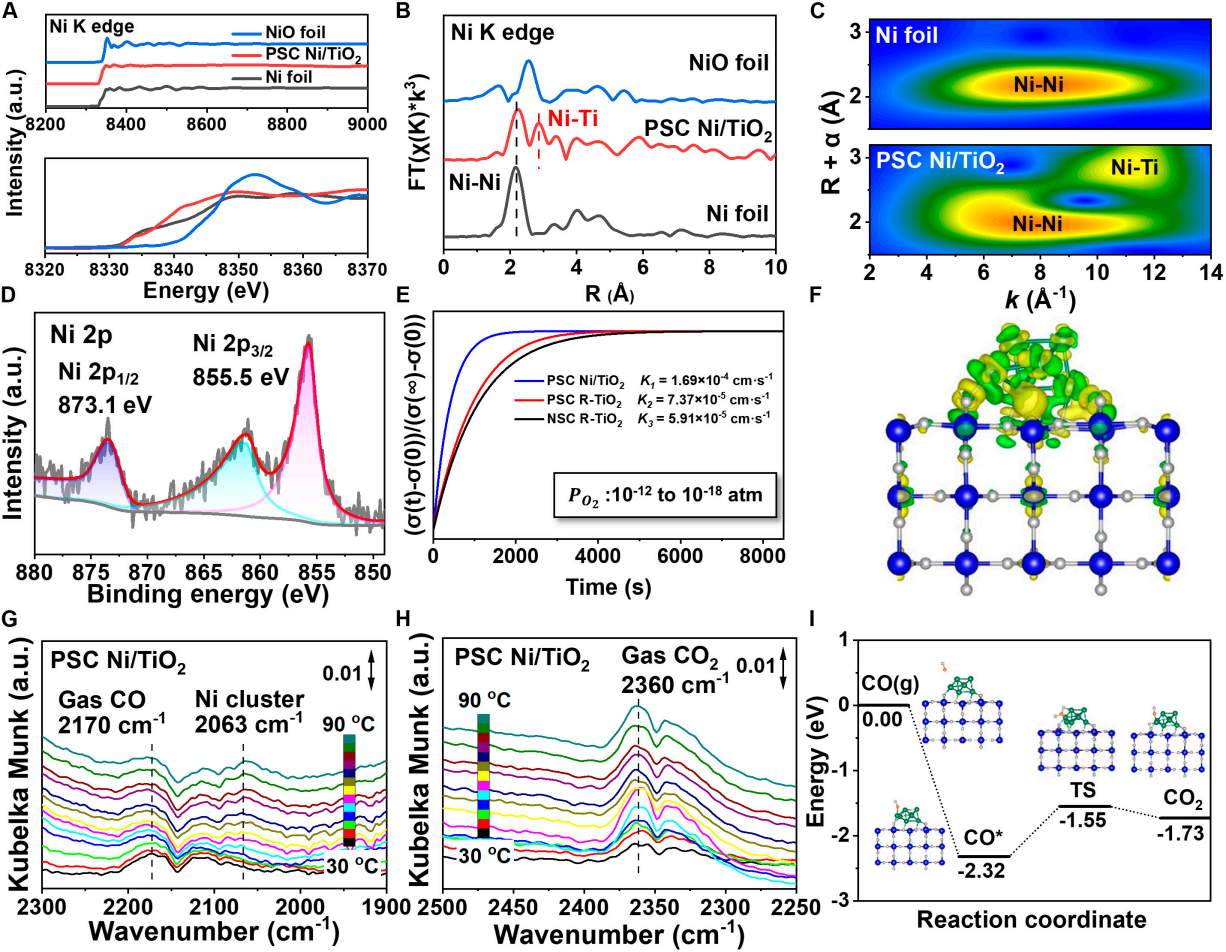
Characterization of PSC Ni/TiO_2_. (A) XAFS spectra (up) and XANES spectra (down) of PSC R-TiO_2_ loading with 0.48 wt % Ni clusters. (B) EXAFS spectra of PSC R-TiO_2_ loading with 0.48 wt % Ni clusters. (C) The 2-dimensional wavelet transform EXAFS spectra of standard Ni foils, NiO foil, and PSC R-TiO_2_ loading with 0.48 wt % Ni clusters. (D) XPS spectra of PSC Ni/TiO_2_. (E) Surface oxygen exchange coefficient of PSC Ni/TiO_2_, PSC R-TiO_2_, and polycrystal R-TiO_2_. (F) Charge density difference of PSC Ni/TiO_2_. The accumulation and depletion of electrons are represented by the yellow and green regions, respectively. Isosurface value = 0.003 e/bohr^3^. (G and H) In situ FTIR of CO oxidation with air using PSC Ni/TiO_2_ monoliths. (I) Potential energy of CO on Ni/TiO_2_ surface (gray is carbon, blue is titanium, green is nickel, and red is oxygen).

The EELS results for PSC Ni/TiO_2_ in Fig. 4F and G exhibit peaks similar to those of PSC R-TiO_2_ at 460 and 465 eV. Additionally, a peak is observed at 855 eV, which corresponds to the L_3_ edge of Ni [35]. This observation demonstrates the successful deposition of Ni clusters onto the surface of PSC R-TiO_2_. The x-ray absorption fine structure (XAFS) and x-ray absorption near edge structure (XANES) are illustrated in Fig. 5A [36,37]. Furthermore, the comparable edge energy observed between PSC Ni/TiO_2_ and conventional Ni foil indicates a favorable metallic state [11,38–40], aligning with the XPS results.

To gain a deeper understanding of the local structure of the PSC Ni/TiO_2_ monoliths, systematic Ni K-edge extended XAFS (EXAFS) data were analyzed using the Athena-Artemis software, as shown in Fig. 5B and C and Fig. S14. The Fourier-transformed EXAFS (FT-EXAFS) spectra of the Ni/TiO_2_ interface system exhibit a prominent peak at 2.22 Å, primarily attributed to the Ni–Ni shell [41–43]. The results of our study indicate that the Ni clusters in PSC Ni/TiO_2_ predominantly exist in a metallic state rather than as NiO. The WT-EXAFS diagram effectively demonstrates the distinct characteristics of standard Ni foil and PSC Ni/TiO_2_, as depicted in Fig. 5C. The highest signal peak (8.2 Å^−1^; 2.2 Å) clearly indicates the presence of a Ni–Ni shell, while the minor signal peak (11.4 Å^−1^; 2.8 Å) corresponds to the existence of a Ni–Ti shell. This provides compelling evidence for the interaction between Ni clusters and TiO_2_ in PSC Ni/TiO_2_. The fitting results of EXAFS are shown in Table S1.

The ALD deposition facilitates the formation of clustered Ni species, which are evenly distributed within the PSC R-TiO_2_ crystals. Figure 5D is the XPS map of Ni clusters deposited on PSC R-TiO_2_ by ALD, which shows the chemical valence states of Ni in PSC Ni/TiO_2_ monoliths. Among them, 855.5 and 873.1 eV belong to Ni 2p_3/2_ and Ni 2p_1/2_, respectively, which is similar to the peak value of standard Ni [44]. This indicates that Ni deposited in PSC R-TiO_2_ mainly exists in the form of zero-valent Ni. The relationship between the surface lattice oxygen exchange coefficient and the surface Ni cluster load content is illustrated in Fig. 5E. The PSC exhibits a surface oxygen exchange coefficient approximately 24.7% higher than that of the nonporous single crystal (NSC) TiO_2_. Moreover, the introduction of the PSC Ni/TiO_2_ interface system leads to an additional increase of about 129.3% in the surface oxygen exchange coefficient. The significant increase in the oxygen exchange coefficient demonstrates that the local Ni/TiO_2_ interface system can greatly enhance the efficiency of oxygen transfer, thereby facilitating the oxidation reaction. The Raman map of the prepared crystal is shown in Fig. S15, where the peaks at 114.7, 445.2, and 610.9 cm^−1^ correspond to the B_1g_, E_g_, and A_1g_ modes of PSC R-TiO_2_, respectively [45]. Notably, the vibration characteristics of PSC R-TiO_2_ at these specific peaks remain unaffected after loading Ni clusters, thereby providing further evidence for the preservation of rutile structure in PSC Ni/TiO_2_. The state density of the sample is illustrated in Figs. S16 and S17. Before constructing the Ni/TiO_2_ interface system, the state density of PSC R-TiO_2_ near the Fermi level approaches zero, whereas after incorporating Ni clusters, Ti and Ni substantially contribute electrons near the Fermi level, thereby greatly enhancing its electrical conductivity and catalytic properties as well as sample activity. By calculating the difference between Ni/TiO_2_ and TiO_2_, we have generated a differential charge density diagram for PSC R-TiO_2_ and PSC Ni/TiO_2_, which is presented in Fig. 5F. The differential charges of PSC R-TiO_2_ and PSC Ni/TiO_2_, as calculated by DFT electron transfer within the interface system of Ni/TiO_2_, occur from Ni to TiO_2_ predominantly toward oxygen vacancy sites on the surface.

**Fig. 6.**
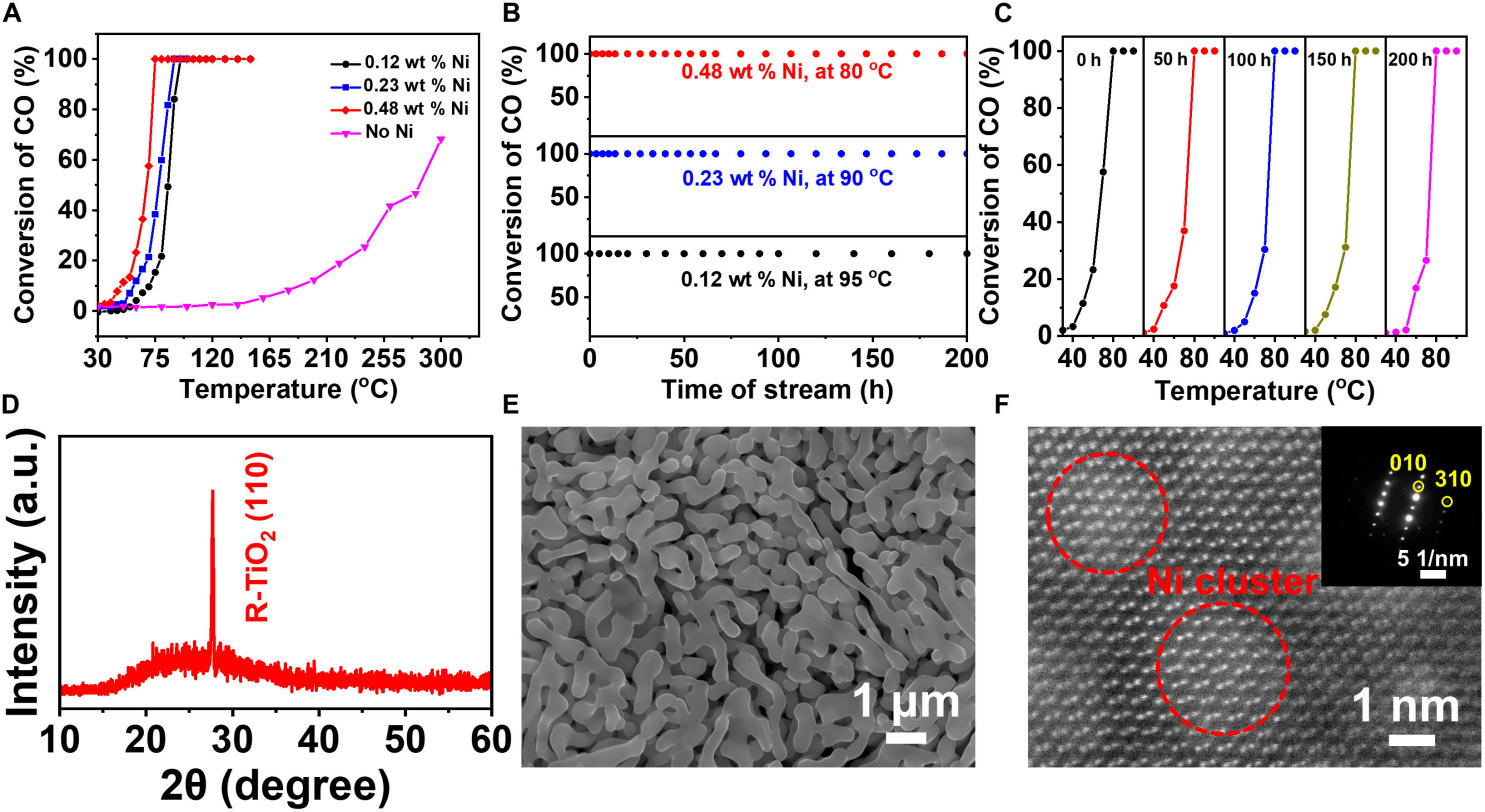
Catalytic performance for CO oxidation with air of PSC (110) Ni/TiO_2_. (A) CO oxidation of PSC R-TiO_2_ loading Ni clusters with different contents. (B) Stability test of PSC Ni/TiO_2_ monoliths. (C) Dynamics of CO oxidation at 0 to 200 h using PSC R-TiO_2_ loading with 0.48 wt % Ni clusters. (D) XRD patterns of PSC (110) TiO_2_ loading with 0.48 wt % Ni clusters after operation of 200 h. (E) SEM image of PSC (110) TiO_2_ loading with 0.48 wt % Ni clusters after operation of 200 h. (F) Cs-HRTEM image and SAED pattern of PSC Ni/TiO_2_ after operation of 200 h.

This confirms that Ni clusters facilitate oxygen vacancy formation in Ni/TiO_2_ interface systems. Investigation of the CO trapping capability of PSC Ni/TiO_2_ was conducted using in situ Fourier transform infrared spectroscopy (FTIR), as depicted in Fig. 5G and H. During the temperature transition from 30 to 90 °C, we observed the peaks of 2,063 cm^−1^, which is the absorption peak of CO captured by Ni clusters. The absorption peak of gas-phase CO and gas-phase CO_2_ are 2,170 and 2,360 cm^−1^, respectively [46]. The adsorption of CO occurs on Ni clusters, followed by its catalytic conversion to CO_2_. The results demonstrate an upward trend in the signal peak of CO adsorption by Ni clusters in PSC Ni/TiO_2_ with increasing temperature, as illustrated in Fig. 5G and H. The simultaneous increase in temperature is accompanied by a noticeable upward trend in the signal peak of gas CO_2_. The observation provides compelling evidence for the exceptional catalytic oxidation performance of PSC Ni/TiO_2_. The transition state of CO oxidation at the PSC Ni/TiO_2_ interface was determined using density functional theory (DFT), as illustrated in Fig. 5I. The adsorption energy of CO on the Ni/TiO_2_ interface structure is approximately 2.32 eV, while the energy barrier for the reaction between lattice oxygen connected with the Ni cluster and CO to form carbon dioxide is around 0.77 eV, indicating a high likelihood for CO oxidation and interaction with lattice oxygen at the interface. The catalytic performance of PSC Ni/TiO_2_ with (110) facet for CO oxidation in air is shown in Fig. 6A and Fig. S18. At low temperature ranging from 30 to 140 °C, PSC R-TiO_2_ without Ni exhibits low enhancement on the CO oxidation reaction. At high temperature of 300 °C, it exhibits discernible catalytic activity, with a conversion rate of ~68.18% observed for the oxidation of CO. In contrast, the PSC R-TiO_2_ with the Ni/TiO_2_ interface system exhibits superior performance by achieving a CO conversion rate up to ~6.62% at near room temperature and complete CO oxidation at 80 °C. Additionally, testing the sample loading with 0.60 wt % Ni did not notably enhance its performance (complete CO oxidation at 75 °C). Furthermore, we conducted additional tests on samples with higher loads; however, results indicated minimal improvement in catalytic oxidation performance upon increasing loading capacity. TiO_2_ supported by varying Ni contents exhibits a consistent enhancement trend at low temperatures, with the catalytic CO oxidation performance gradually improving as the temperature increases. Furthermore, we introduced additional data of Ni/TiO_2_ with higher content. When the Ni cluster content of PSC Ni/TiO_2_ exceeds 0.48 wt %, the catalytic oxidation performance of PSC Ni/TiO_2_ has no marked improvement.

The durability test results of PSC Ni/TiO_2_ containing 0.48 wt % Ni are presented in Fig. 6B, demonstrating the remarkable capability of PSC Ni/TiO_2_ to maintain a consistent conversion rate of 100% even after continuous operation at 80 °C for 200 h. Figure 6C demonstrates the dynamics of CO oxidation at 0 to 200 h using PSC Ni/TiO_2_ loading with 0.48 wt % Ni. Remarkably, the catalysis activity of PSC Ni/TiO_2_ loading with 0.48 wt % Ni shows negligible deterioration after multiple operational cycles. The results demonstrate that our Ni cluster/TiO_2_ interface system effectively activates the surface lattice oxygen, leading to a dual activation of both the surface lattice oxygen and CO molecules. The incorporation of the Ni cluster/TiO_2_ interface system substantially enhances the catalytic performance of PSC R-TiO_2_ for CO oxidation while maintaining long-term stability during prolonged reaction. The characteristics of PSC Ni/TiO_2_ after a 200-h CO oxidation reaction are illustrated in Fig. 6D and F and Fig. S19. The SEM, Cs-HRTEM, SAED, and XRD analyses reveal that the PSC Ni/TiO_2_ monoliths retain excellent monocrystalline properties even after prolonged reaction time. The XPS spectra results presented in Fig. S19B to D demonstrate that the valence states of Ti and O elements in PSC Ni/TiO_2_ remain unchanged before and after the CO oxidation reaction of 200 h, with only a minor fraction (5.99%) of Ni being converted to its divalent state. The aforementioned characteristics suggest that PSC Ni/TiO_2_ exhibits excellent structural and chemical stability.

## Conclusion

In conclusion, we have successfully fabricated 1-cm-scale PSC rutile TiO_2_ monoliths using a solid–solid phase transformation strategy. The single-crystalline solid phase framework with long-range ordered lattice provides structural advantages for creating the well-defined interface system, while the 3-dimensional interconnected pores enhance permeation and species diffusion. We constructed the metal–oxide interface by uniformly confining atomic-layer Ni clusters on the lattice surface of PSC TiO_2_, which enhanced the catalytic oxidation performance of CO. We have confirmed that the PSC Ni/TiO_2_ catalyst can efficiently capture CO and activate lattice oxygen at temperatures close to temperature, exhibiting complete catalytic oxidation performance of CO at ~80 °C, while maintaining excellent catalytic activity and stability even after continuous operation for 200 h. The current work serves as a reference for the development of other advanced ternary solid catalysts.

## Materials and Methods

### Crystal growth of PSC TiO_2_

The KTP crystals are precisely cut and meticulously polished along the *a* axis, *b* axis, and *c* axis to prepare a mother phase measuring 10 mm × 10 mm × 0.5 mm in dimensions for subsequent growth of PSC TiO_2_ monoliths. The growth of PSC R-TiO_2_ monolith was conducted within a chemical vapor deposition (CVD) system equipped with a precise pressure controller and mass flowmeter. The PSC TiO_2_ monoliths were prepared under controlled atmospheric conditions at pressures ranging from 50 to 400 torr (5% O_2_/Ar, flow rates between 50 and 200 sccm with purity of 6 N) and temperatures ranging from 900 to 1,000 °C for durations of 100 to 200 h. A simplified model was used to determine the lattice mismatch between the epitaxial growth layer and substrate.

### Characterization

XRD analysis using Cu–Kα radiation (Mniflex 600) was employed to determine the phase structure and facet orientation of the samples. Field emission SEM (FESEM; SU8010) imaging at an accelerating voltage of 10 kV was utilized to capture images of nanoparticles exhibiting a porous surface morphology. FIB technique (Helios 650, Zeiss Auriga) was employed for sample preparation in order to observe the samples under TEM (FEI Titan3 G2 60-300). Field emission TEM (FETEM) and HRTEM were applied to analyze the microstructures of the nanoparticles. In situ FTIR spectroscopy was performed using a Bruker VERTEX 70 IR spectrometer. We use ALD strategy to construct the isolated Ni clusters/TiO_2_ sites (D100-4882, China). EXAFS measurements were conducted to analyze the coordination features of the samples. Raman spectroscopy was used to determine the structures of both precursors and products, employing Raman spectrometry (LabRAMHR, Horiba J.Y.) for further phase characterization. Inductively coupled plasma (ICP; Avio220Max) is employed for the quantitative determination of Ni content in PSC Ni/TiO_2_.

### Theoretical calculation

The density functional theory (DFT) calculations were performed using the Vienna Ab initio Simulation Package (VASP) [47,48], employing the generalized gradient approximation (GGA) Perdew–Burke–Ernzerhof (PBE) functional [49] to account for electron exchange and correlation. The projector-augmented plane wave (PAW) [50,51] potentials were utilized to describe the interaction between core and valence electrons, employing a plane wave basis set with a kinetic energy cutoff of 500 eV. Partial occupancies of the Kohn−Sham orbitals were allowed using the Gaussian smearing method with a width of 0.05 eV. Self-consistency was achieved when the change in electronic energy was less than 10^−5^ eV. Geometry optimization was considered convergent when the change in force was smaller than 0.02 eV/Å.

### Catalytic test

The deposition of PSC TiO_2_ monoliths loading Ni cluster with different contents was achieved via the ALD method by exposing them at 120 °C. PSC Ni/TiO_2_ quadrate monoliths, with a porosity of 70.0%, were introduced into a microtubular quartz reactor with an identical diameter of 5 mm. The CO catalytic testing was conducted using a securely positioned 100-mg PSC Ni/TiO_2_ catalyst at the center of the reactor under atmospheric pressure. The CO catalytic unit was enclosed by a temperature-sensitive holding furnace, and the program temperature rose from 25 to 150 °C at a speed of 2 °C min^−1^, with each test temperature point being stabilized for 30 min before testing. The reaction gas, consisting of equilibrium mixtures containing 1% CO and 29% air in Ar, was fed to the reactor at a total flow rate of 40 sccm. Online analysis of reaction performance was carried out using gas chromatography–mass spectrometry (GCMS-QP2010 SE) equipped with flame ionization detector and thermal conductivity detector (Shimadzu, GC-2014).

## Data Availability

All data are available in the manuscript or Supplementary Materials or from the author.
