# Supplementary material for: Engineering Active Interfaces on the Surface of Porous Single-Crystalline TiO2 Monoliths for Enhanced Catalytic Activity and Stability
Source: Research (Wash D C). 2025 Jan 14;8:0579. doi: 10.34133/research.0579 (PMC11729270; doi:10.34133/research.0579)
Supplement: Supplementary 1 — Figs. S1 to S19 Table S1 [file research.0579.f1.docx]

**Front Matter**

Title

**Engineering Active Interfaces on the Surface of Porous Single-Crystalline TiO_2_ Monoliths for Enhanced Catalytic Activity and Stability**

Short title: Interfaces on TiO_2_ Monoliths for Enhanced Catalytic Activity

**Authors**

Huang Lin ^1,2,3,4^, Cong Luo^1,2,4^, Fangyuan Cheng ^1,2,3,4*^ and Kui Xie ^1,2,3,4,5*^

**Affiliations**

^1^ Key Laboratory of Design & Assembly of Functional Nanostructures, Fujian Institute of Research on the Structure of Matter, Chinese Academy of Sciences, Fuzhou, Fujian, 350002, China.

^2^ Fujian Science & Technology Innovation Laboratory for Optoelectronic Information of China, Fuzhou, Fujian, 350108, China.

^3^ University of Chinese Academy of Sciences, Beijing, 100049, China.

^4^ Fujian College, University of Chinese Academy of Sciences, Fuzhou, Fujian, 350108, China.

^5^ School of Mechanical Engineering, Shanghai Jiao Tong University, Shanghai, 200240, China.

^*^Address correspondence to: [cfy@fjirsm.ac.cn](mailto:cfy@fjirsm.ac.cn) (F.C.) and [xiekui@sjtu.edu.cn](mailto:xiekui@sjtu.edu.cn) (K.X.)


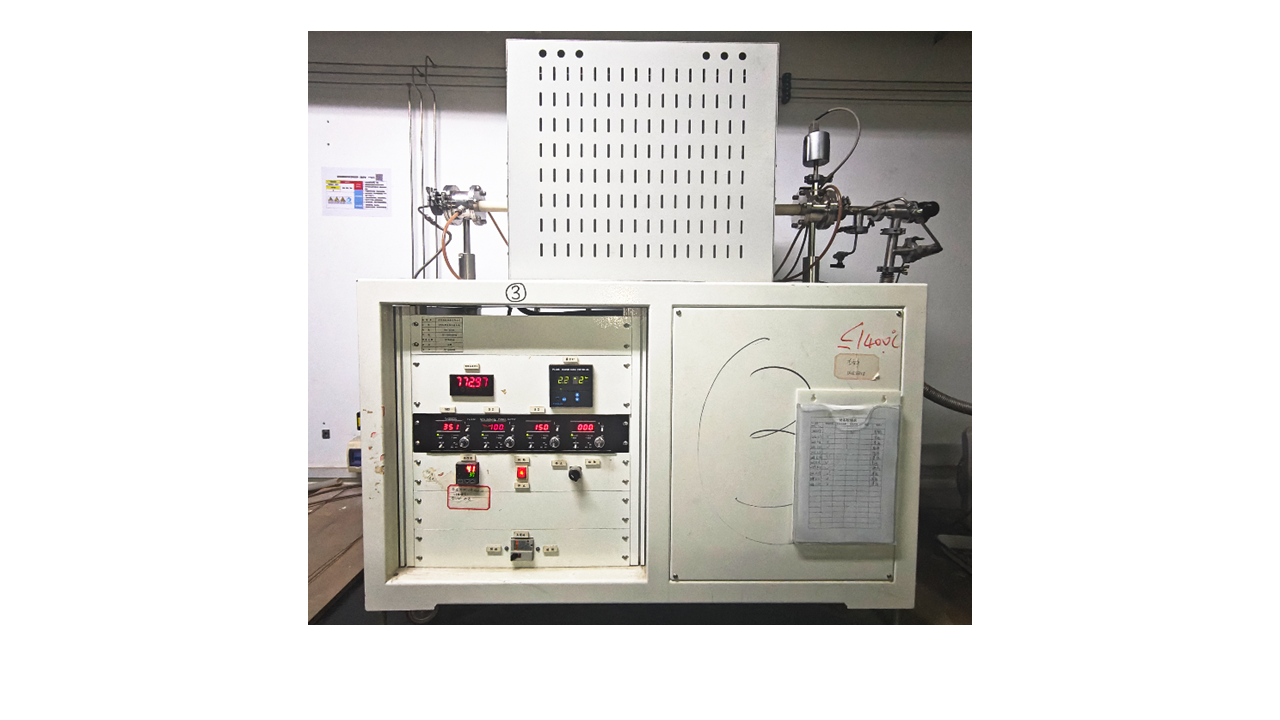


***Fig S1:*** *Experimental setup of preparing PSC TiO_2_ monoliths. Photo of Chemical Vapor Deposition (CVD) system equipped with a precise pressure controller and mass flowmeter.*


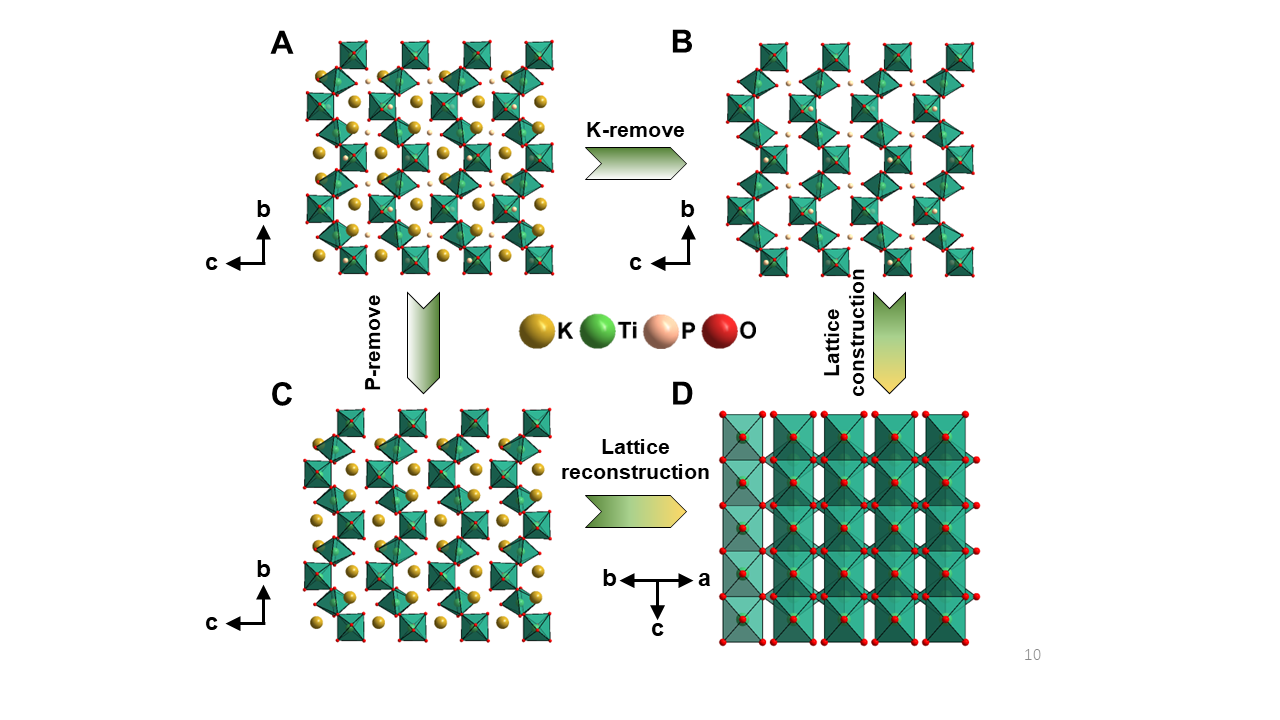


***Fig. S2.*** *Lattice reconstruction of PSC R-TiO_2_.* ***(A)*** *Crystal structure along a-axis KTP.* ***(B)*** *K evaporation channels in a-axis KTP.* ***(C)*** *P evaporation channels in a-axis KTP.* ***(D)*** *Crystal structure of PSC R-TiO_2_.*


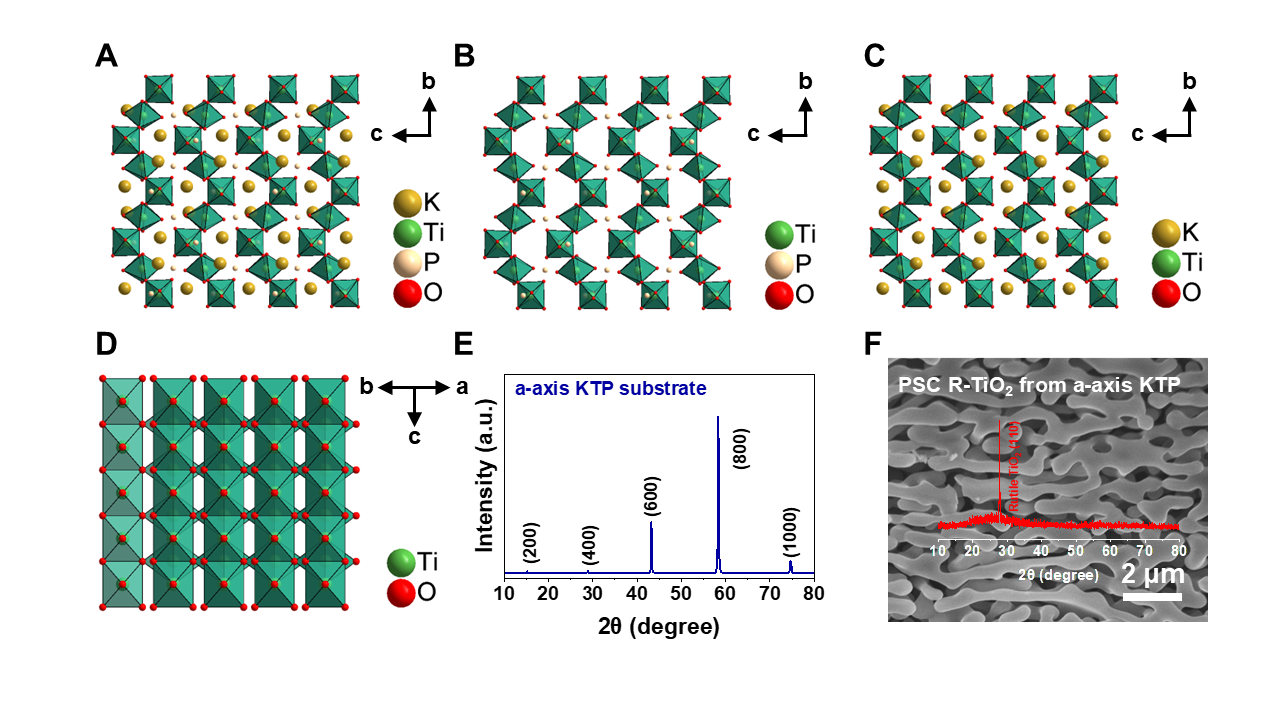


***Fig. S3.*** *Crystal Growth of PSC R-TiO_2_ on a-axis KTP.* ***(A)*** *Crystal structure along a-axis KTP.* ***(B)*** *K evaporation channels in a-axis KTP.* ***(C)*** *P evaporation channels in a-axis KTP.* ***(D)*** *Crystal structure of R-TiO_2_* *(view along 110 axis).* ***(E)*** *XRD pattern of a-axis KTP substrate.* ***(F)*** *XRD pattern and SEM image of PSC R-TiO_2_ grown from a-axis KTP.*


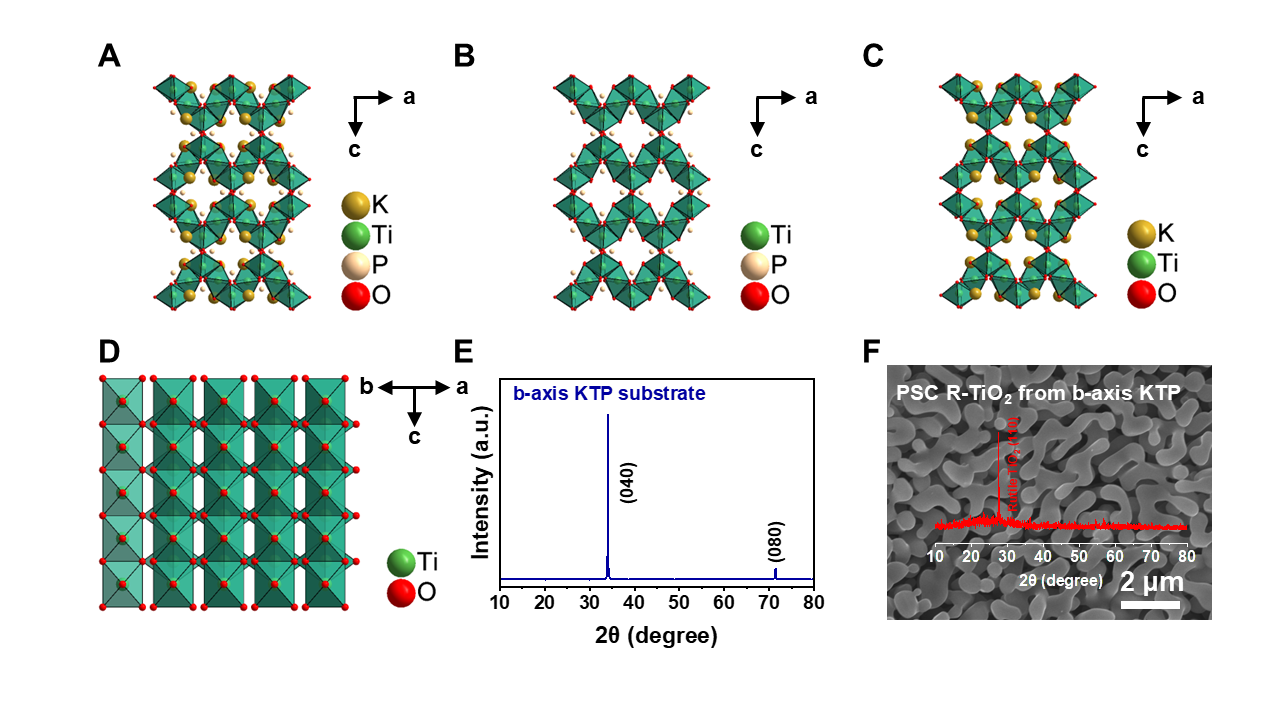


***Fig. S4.*** *Crystal Growth of PSC R-TiO_2_ on b-axis KTP.* ***(A)*** *Crystal structure along b-axis KTP.* ***(B)*** *K evaporation channels in b-axis KTP.* ***(C)*** *P* *evaporation channels in b-axis KTP.* ***(D)*** *Crystal structure of R-TiO_2_ (view along 110 axis).* ***(E)*** *XRD pattern of b-axis KTP substrate.* ***(F)*** *XRD pattern and SEM image of PSC R-TiO_2_ grown from b-axis KTP.*


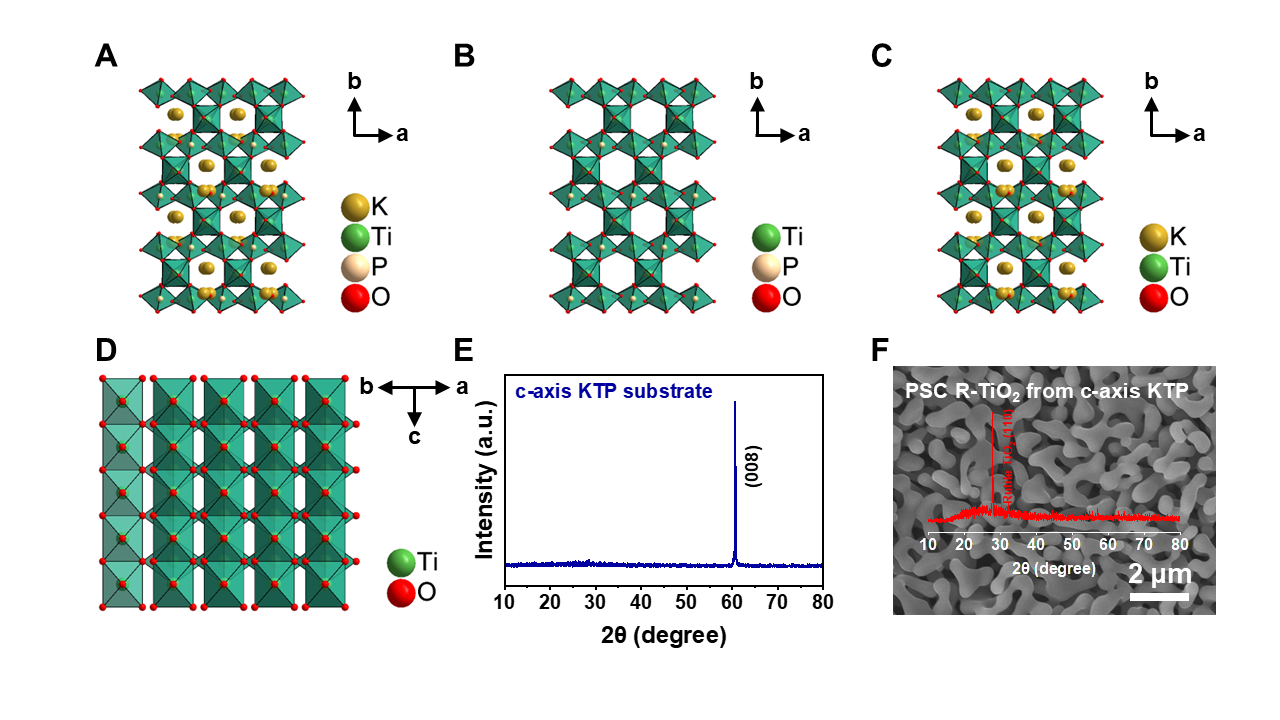


***Fig. S5.*** *Crystal Growth of PSC R-TiO_2_ on c-axis KTP.* ***(A)*** *Crystal structure along c-axis KTP.* ***(B)*** *K evaporation channels in c-axis KTP.* ***(C)*** *P evaporation channels in c-axis KTP.* ***(D)*** *Crystal structure of R-TiO_2_ (view along 110 axis).* ***(E)*** *XRD pattern of c-axis KTP substrate.* ***(F)*** *XRD and SEM image of PSC R-TiO_2_ grown from c-axis KTP.*


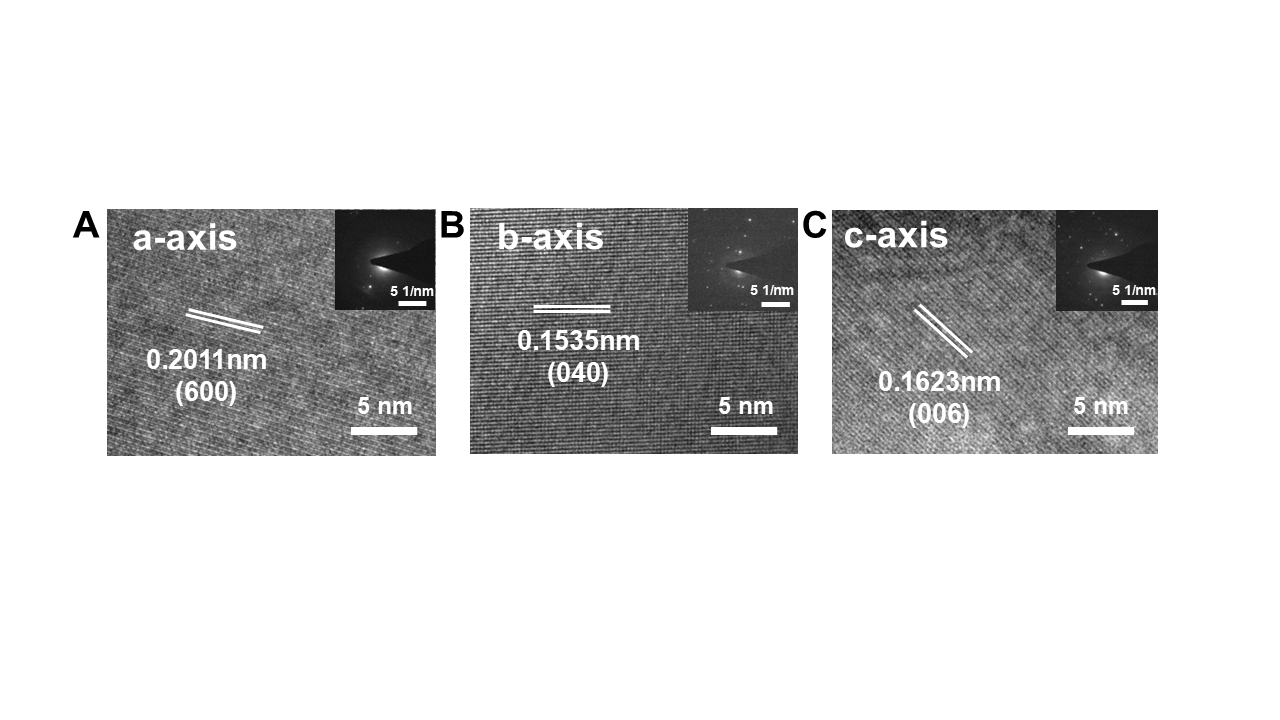


***Fig. S6.*** *Microstructure characterization of KTP.* ***(A)*** *The TEM image and SAED pattern of a-axis KTP.* ***(B)*** *The TEM image and SAED pattern of b-axis KTP.* ***(C)*** *The TEM image and SAED pattern of c-axis KTP.*


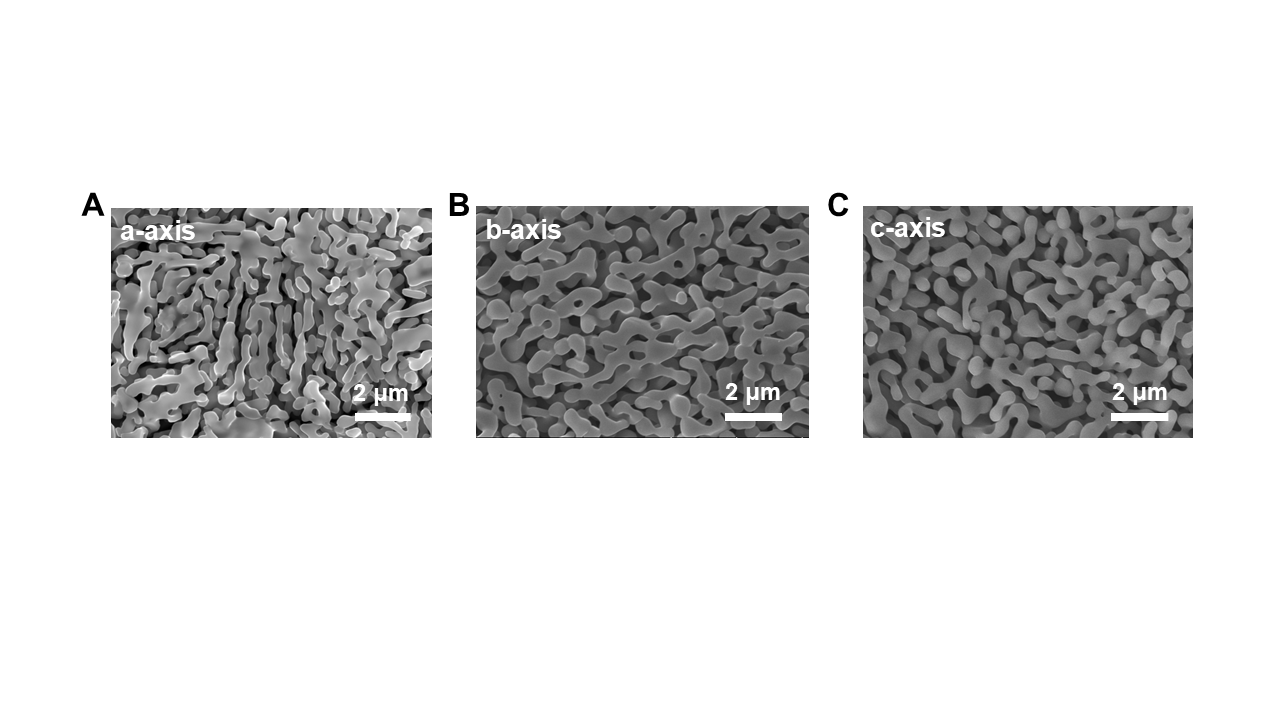


***Fig. S7.*** *SEM characterization of PSC R-TiO_2_.* ***(A)*** *The SEM image of R-TiO_2_ grown from a-axis.* ***(B)*** *The SEM image of R-TiO_2_ grown from b-axis.* ***(C)*** *The SEM image of R-TiO_2_ grown from c-axis.*


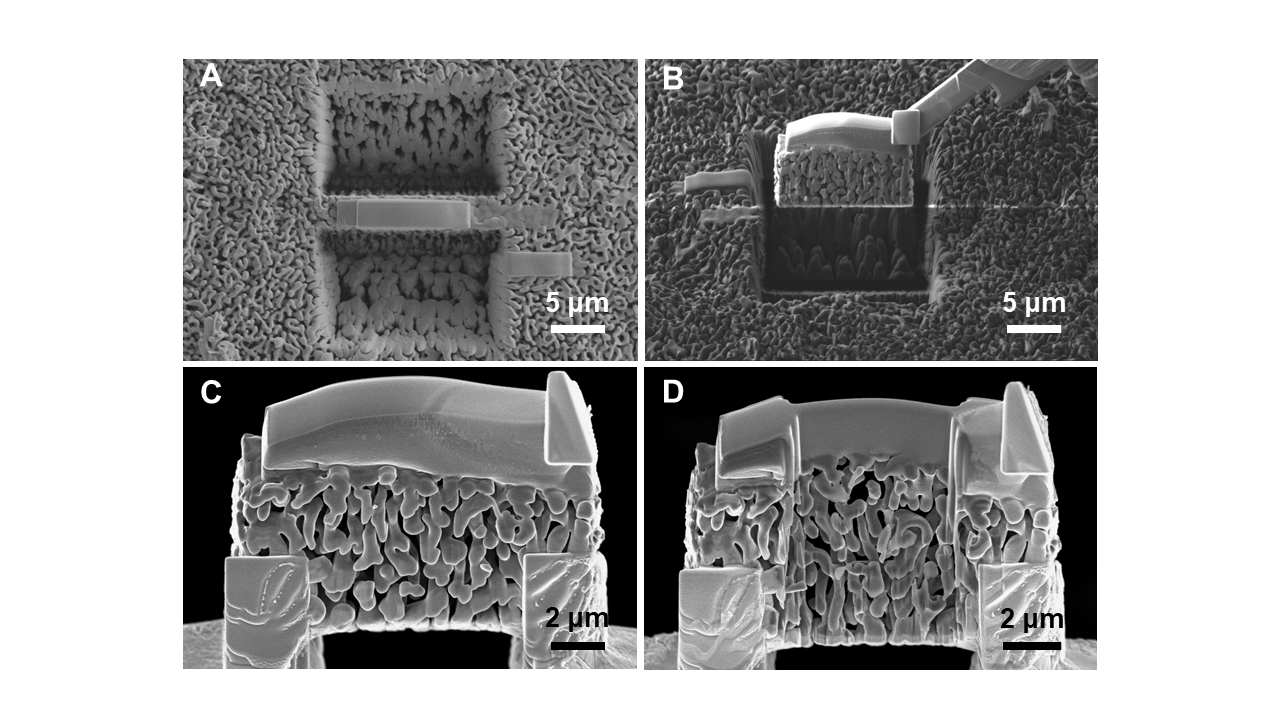


***Fig. S8.*** *FIB preparation process of PSC TiO_2_.*


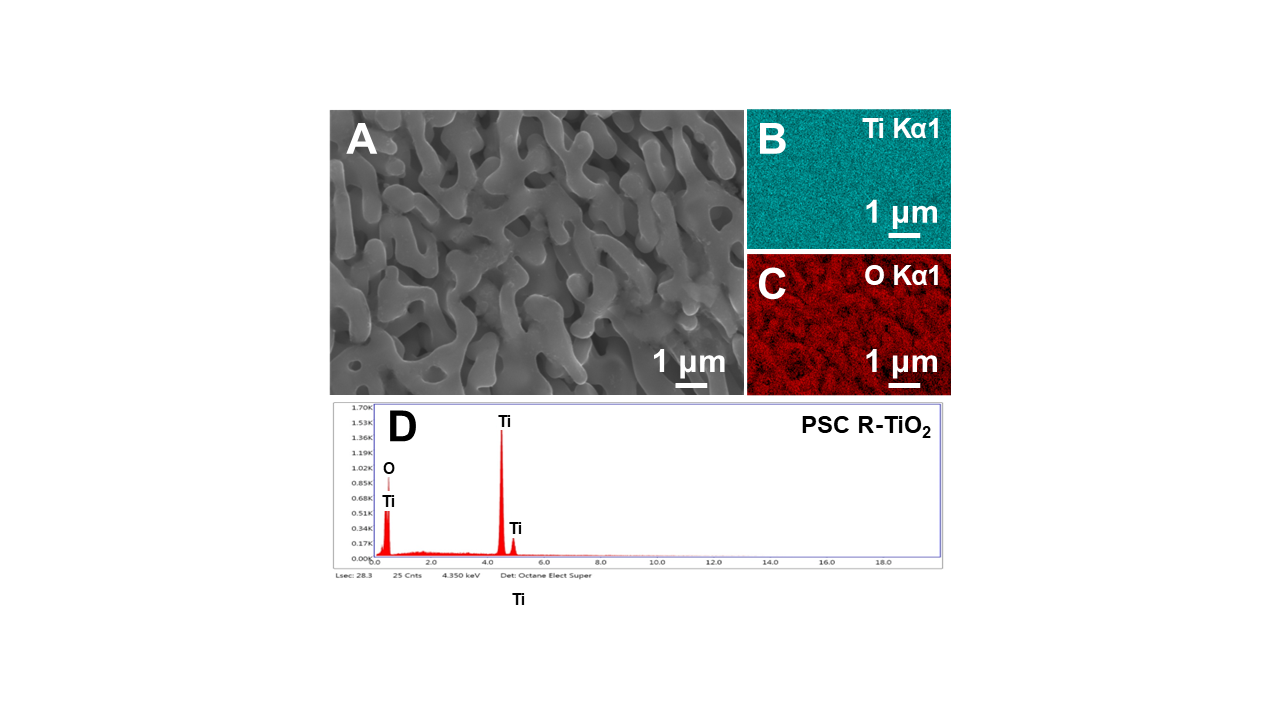


***Fig. S9.*** *Elemental analysis is conducted on PSC R-TiO_2_ monolith.* ***(A)*** *The SEM image of PSC R-TiO_2_.* ***(B and C)*** *The element mapping of PSC R-TiO_2_.* ***(D)*** *the EDS tests of PSC R-TiO_2_.*


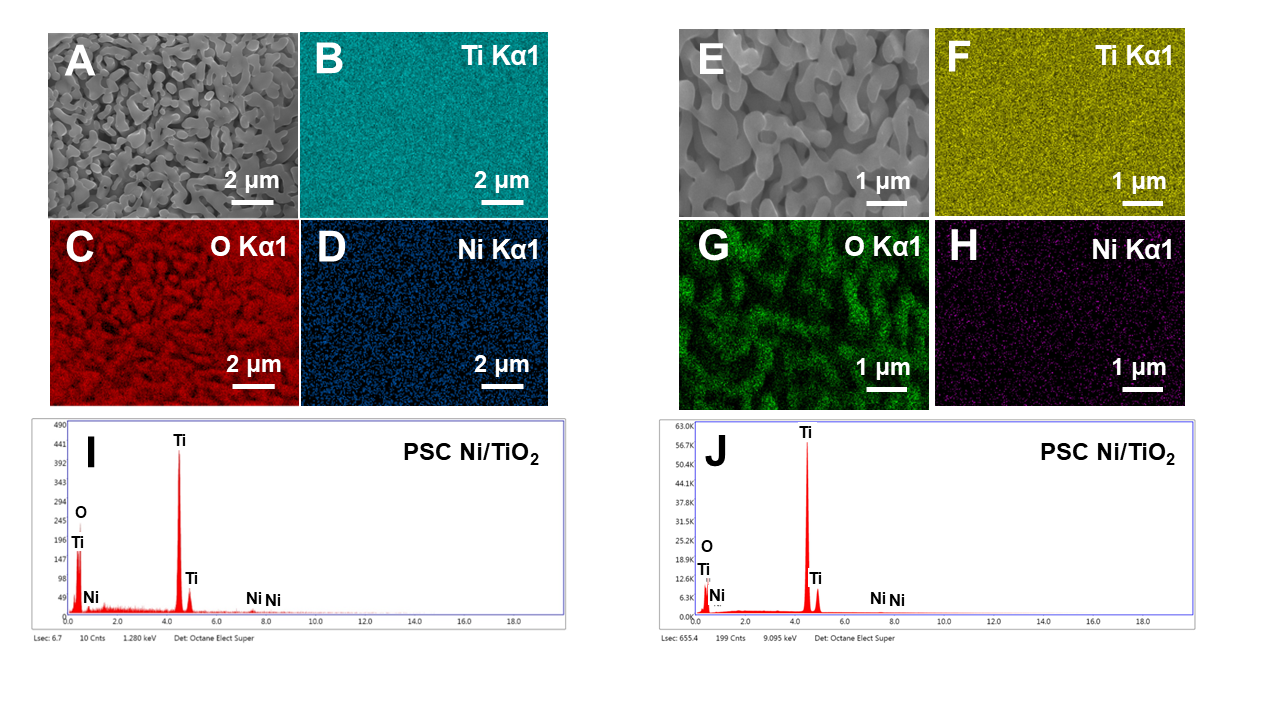


***Fig. S10.*** *The elemental analysis is conducted on PSC R-TiO_2_ monolith loading with Ni clusters.* ***(A to H)*** *The SEM image and the element mapping of PSC Ni/TiO_2_.* ***(I and J)*** *the EDS tests of PSC Ni/TiO_2_.*


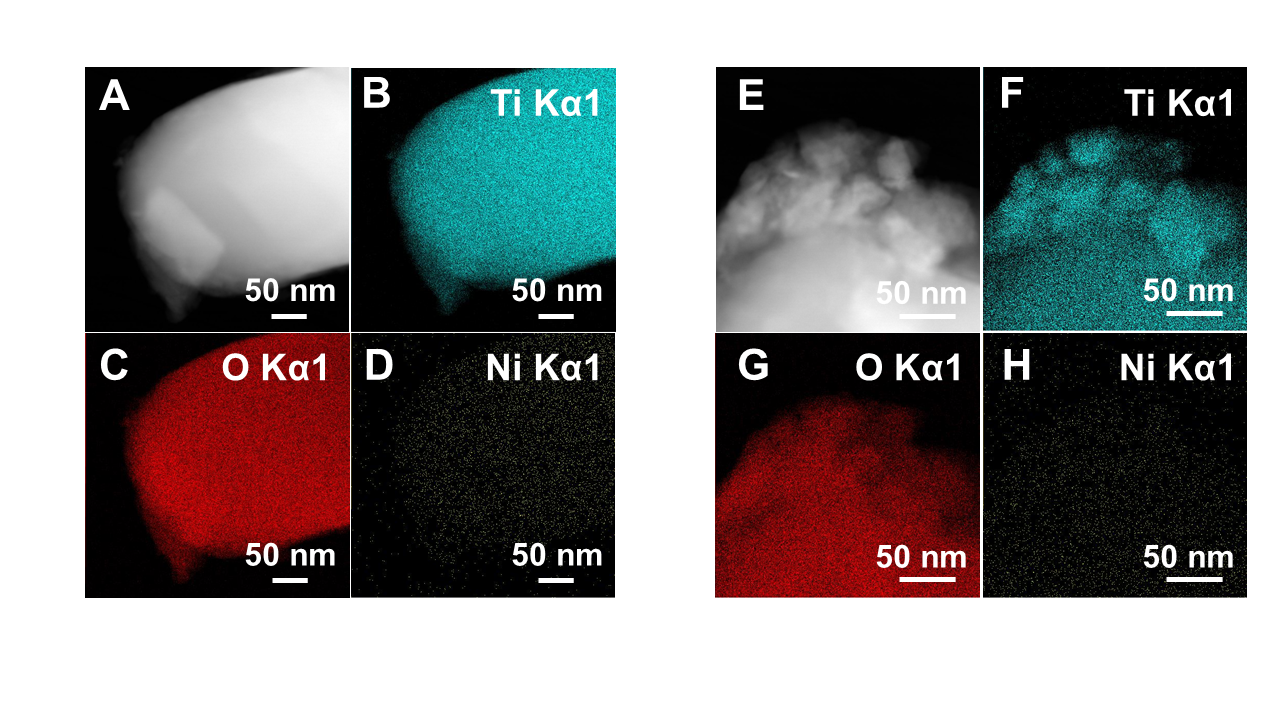


***Fig. S11.*** *The elemental analysis is conducted on PSC R-TiO_2_ monolith loading with Ni clusters.* ***(A to H)*** *The TEM image and the element mapping of PSC Ni/TiO_2_.*


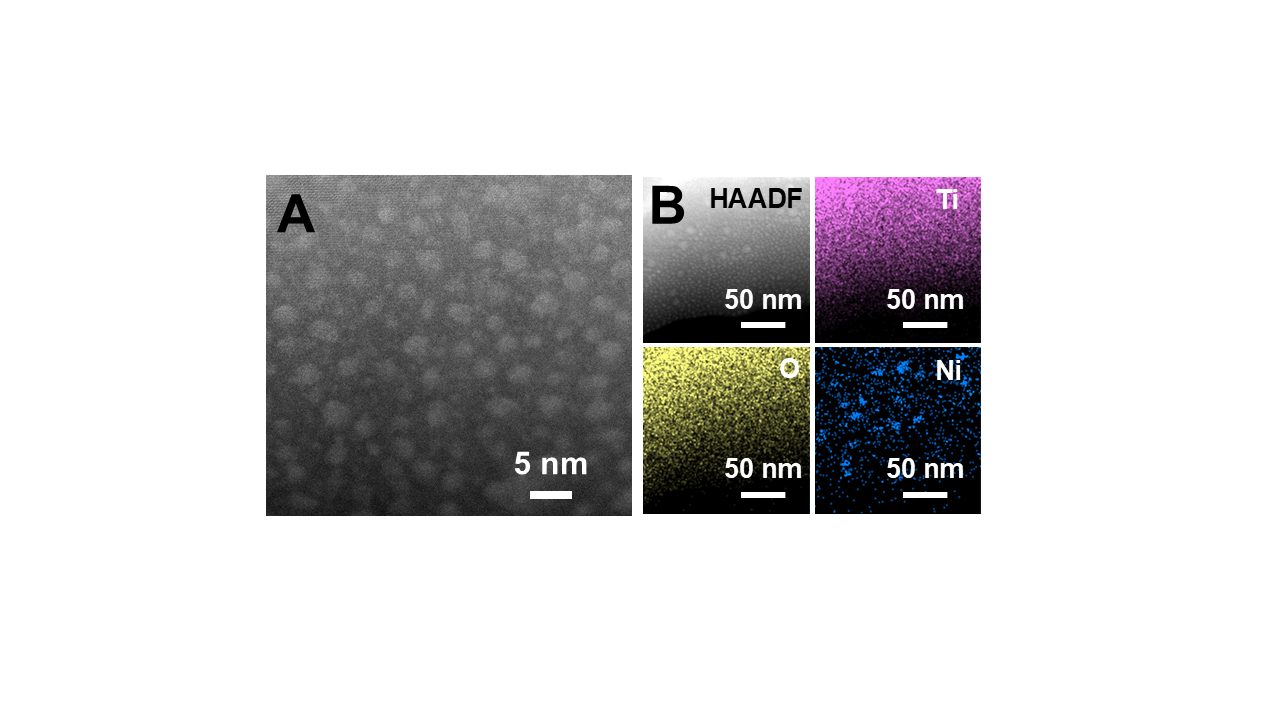


***Fig. S12.*** *The Cs-STEM analysis is conducted on PSC R-TiO_2_ monolith loading with Ni clusters.* ***(A)*** *The Cs-STEM image of PSC Ni/TiO_2_.* ***(B)*** *The HADDF image of PSC Ni/TiO_2_.*


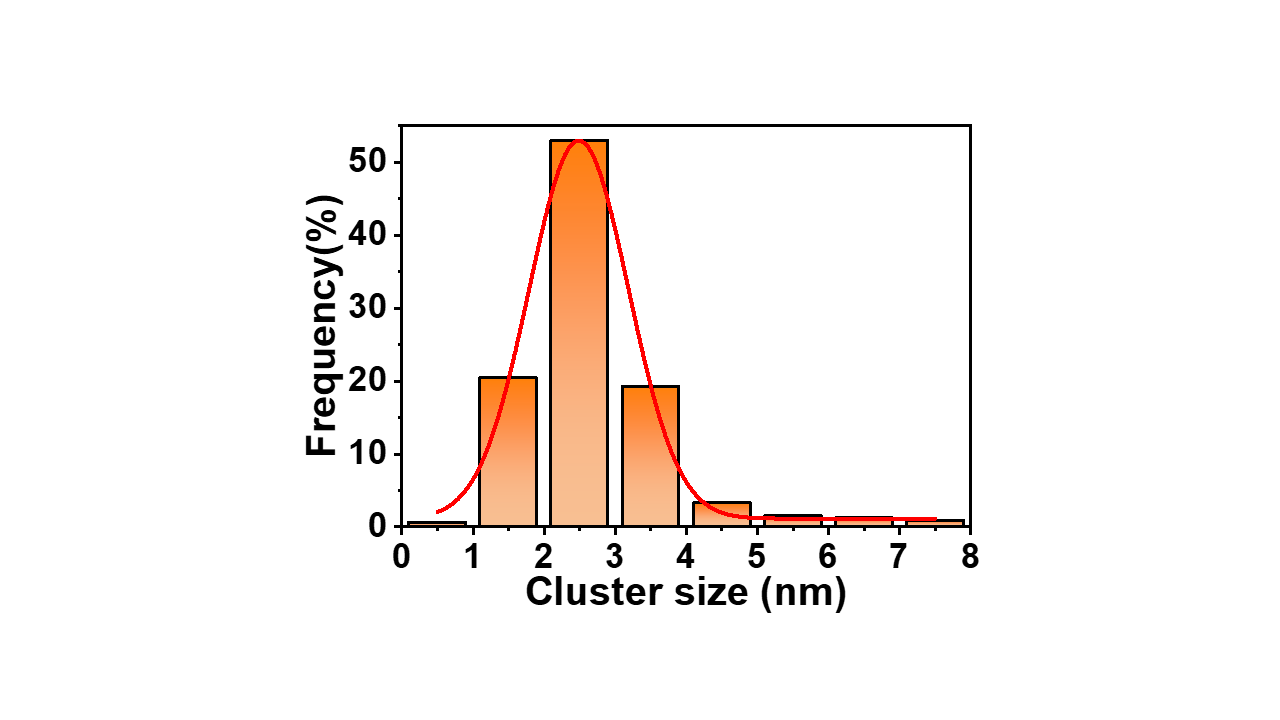


***Fig. S13.*** *The typical size of the Ni cluster in the PSC Ni/TiO_2_.*

*
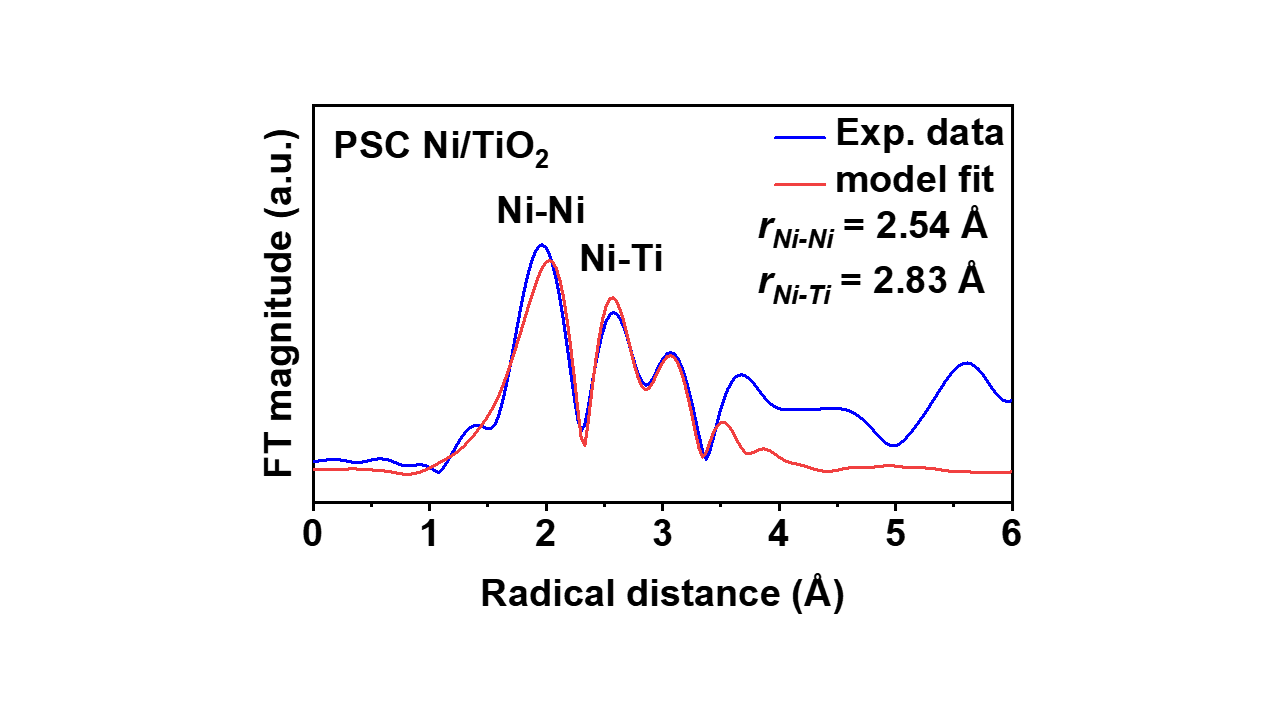
*

***Fig. S14.*** *Fourier transforms of the Ni K-edge EXAFS spectra for the PSC TiO_2_ loading with Ni clusters.*

| **Samples** | **Path** | **CN** | **R (Å)** | **σ^2^ (×10^-3^ Å^2^)** | **ΔE0 (eV)** | **R factor (%)** |
| --- | --- | --- | --- | --- | --- | --- |
| **Ni foil** | Ni-Ni | 12 | 2.48 ± 0.01 | 6.02 ± 0.01 | 6.7 ± 0.4 | 0.19 |
| **NiO** | Ni-Ni | 12 | 2.95 ± 0.01 | 7.04 ± 0.01 | -2.6 ± 0.7 | 0.61 |
|  | Ni-O | 6 | 2.07 ± 0.01 | 5.91 ± 0.01 | -2.6 ± 0.7 |  |
| **PSC Ni/TiO_2_** | Ni-Ni | 3.3 ± 1.7 | 2.54 ± 0.02 | 1.69 ± 0.01 | 5.6 ± 0.6 | 1.12 |
|  | Ni-Ti | 7.3 ± 1.8 | 2.83 ± 0.02 | 1.68 ± 0.01 | 5.6 ± 0.6 |  |

***Table S1.*** *The fitting parameters of Ni K-edge Fourier-filtered k^3^-weighted EXAFS for PSC Ni/TiO_2_.*


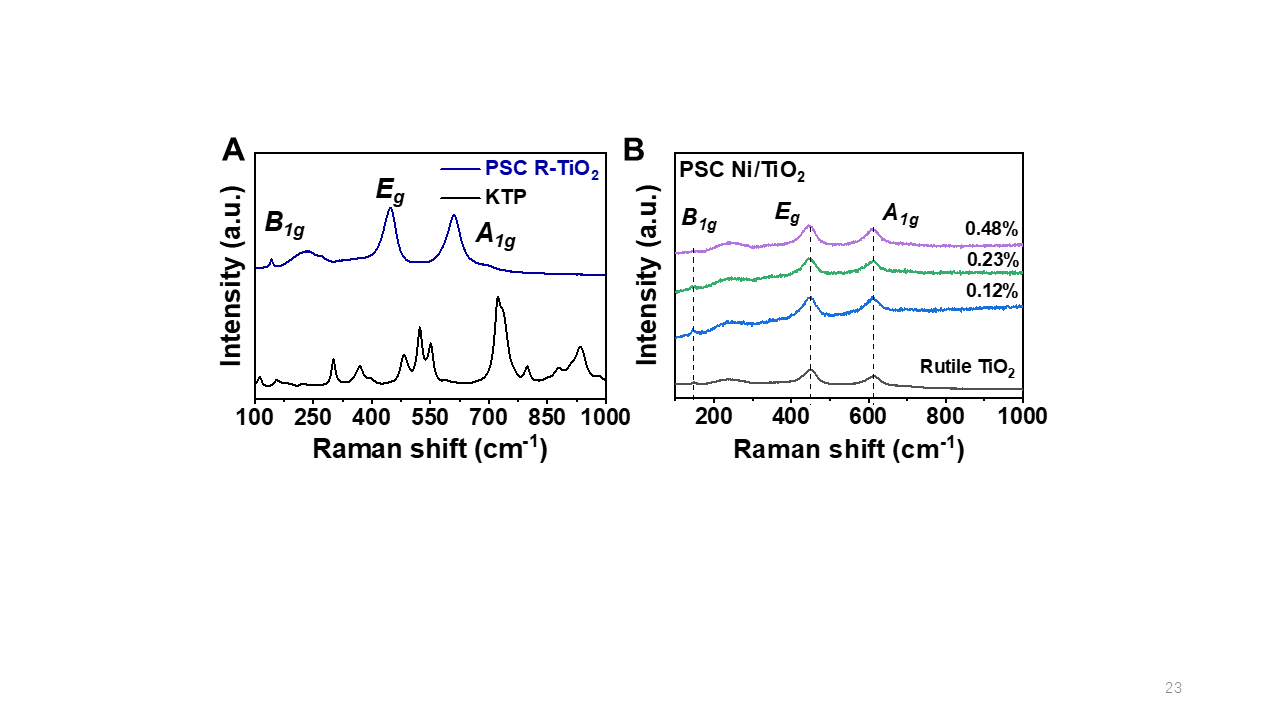


***Fig. S15.*** *Raman spectra of KTP, PSC R-TiO_2_ and PSC Ni/TiO_2_ with excitation lines at 532 nm. (A) Raman spectra of KTP and PSC R-TiO_2_. (B) Raman spectra of R-TiO_2_ loading with Ni clusters.*


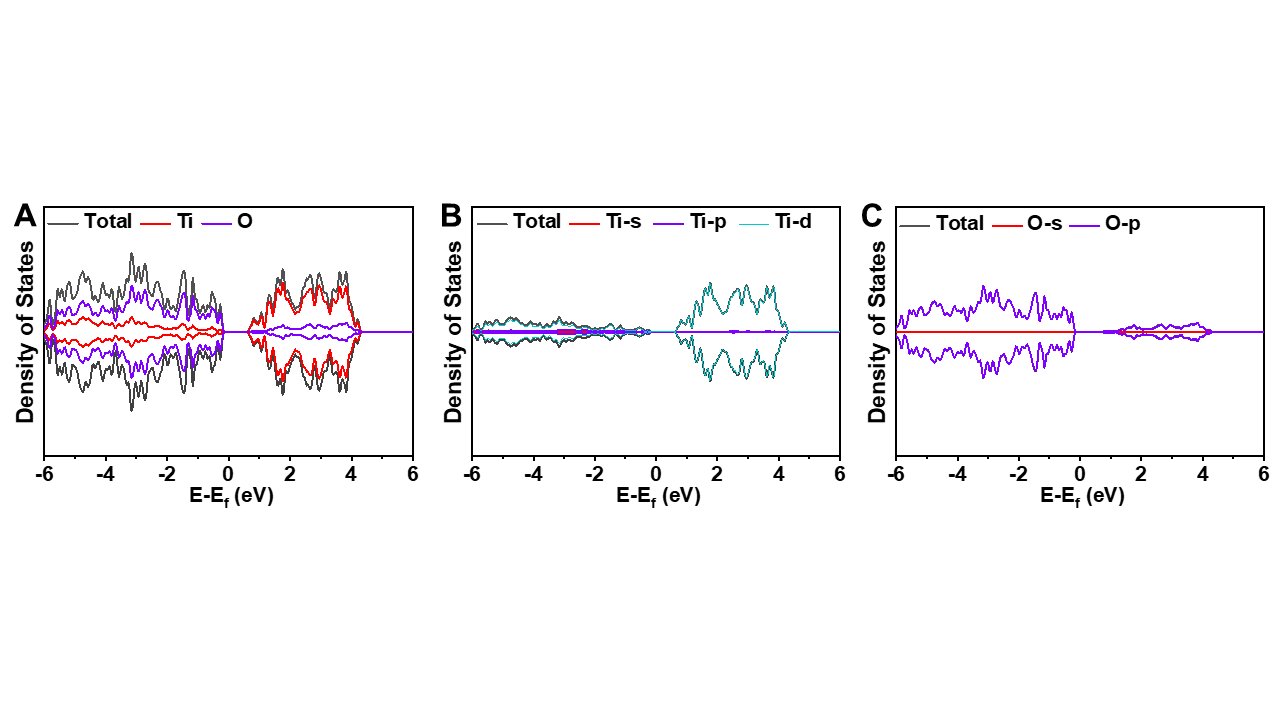


***Fig. S16.*** *Density of state of PSC R-TiO_2_.*


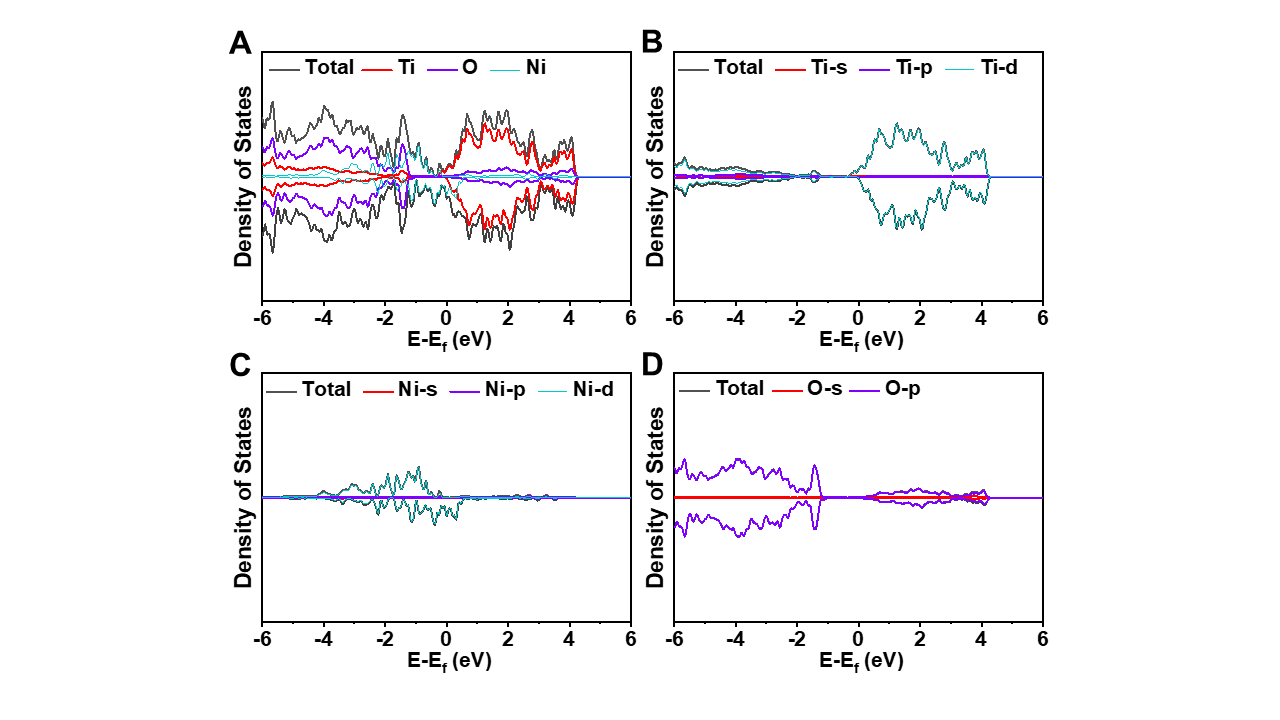


***Fig. S17.*** *Density of state of PSC Ni/TiO_2_.*


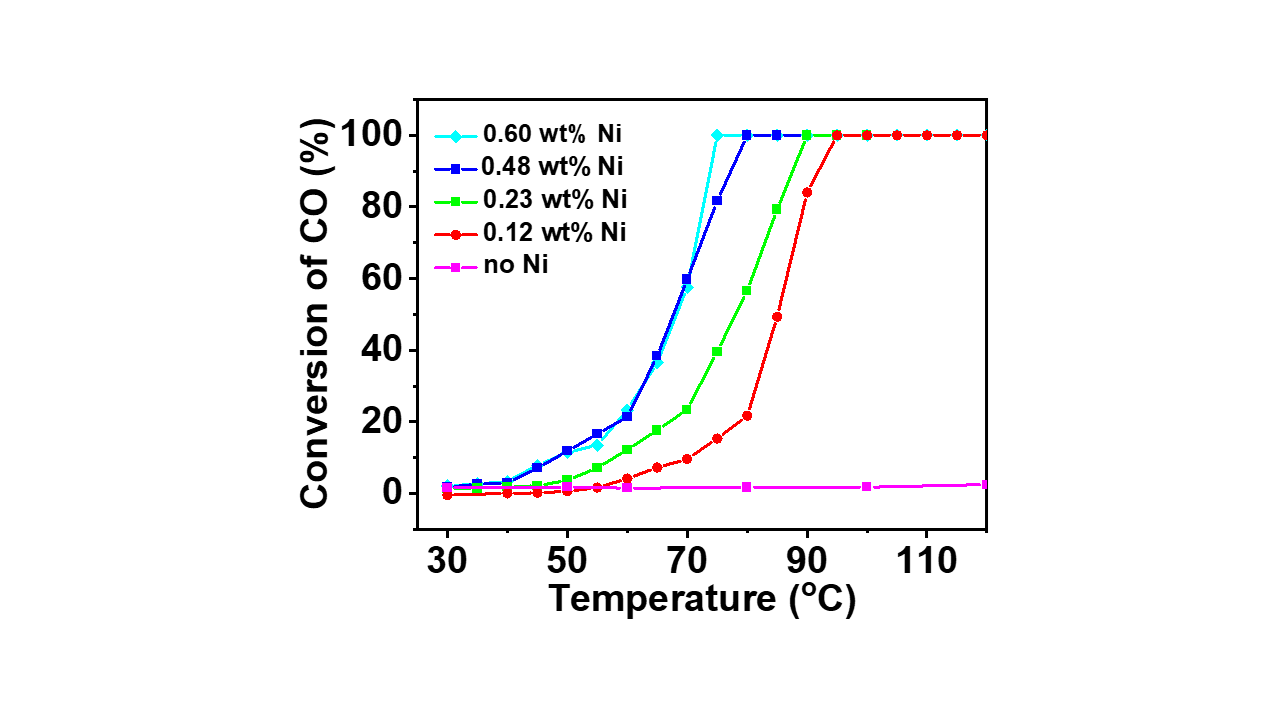


***Fig. S18.*** *CO oxidation of PSC R-TiO_2_ loading Ni clusters with different contents.*


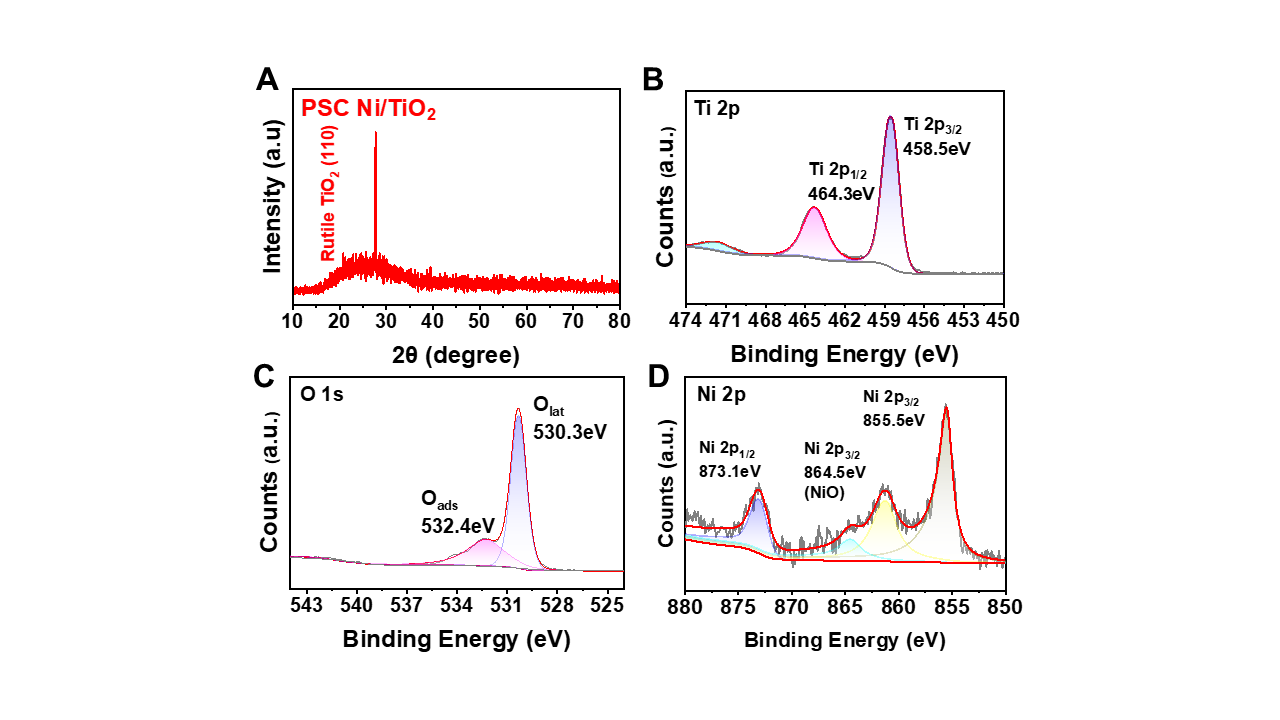


***Fig. S19.*** *Characterization of PSC Ni/TiO_2_ monolith after CO oxidation raction.* ***(A)*** *The XRD spectrum of PSC Ni/TiO_2_ monolith after CO oxidation raction after 200 hours.* ***(B to D)*** *The XPS spectrum of PSC Ni/TiO_2_ monolith after CO oxidation raction after 200 hours.*
